# A monotopic aluminum telluride with an Al=Te double bond stabilized by *N*-heterocyclic carbenes

**DOI:** 10.1038/ncomms10037

**Published:** 2015-11-27

**Authors:** Daniel Franz, Tibor Szilvási, Elisabeth Irran, Shigeyoshi Inoue

**Affiliations:** 1 Institut für Chemie, Anorganische und Analytische Chemie, Technische Universität Berlin, Straße des 17 Juni 135, Sekr. C2, 10623 Berlin, Germany; 2 Department of Inorganic and Analytical Chemistry, Budapest University of Technology and Economics, Szent Gellért tér 4, 1111 Budapest, Hungary

## Abstract

Aluminum chalcogenides are mostly encountered in the form of bulk aluminum oxides that are structurally diverse but typically consist of networks with high lattice energy in which the chalcogen atoms bridge the metal centres. This makes their molecular congeners difficult to synthesize because of a pronounced tendency for oligomerization. Here we describe the isolation of the monotopic aluminum chalcogenide (L^Dip^N)AlTe(L^Et^)_2_ (L^Dip^=1,3-(2,6-diisopropylphenyl)-imidazolin-2-imine, L^Et^=1,3-diethyl-4,5-dimethyl-imidazolin-2-ylidene). Unique features of (L^Dip^N)AlTe(L^Et^)_2_ are the terminal position of the tellurium atom, the shortest aluminum–tellurium distance hitherto reported for a molecular complex and the highest bond order reported for an interaction between these elements, to the best of our knowledge. At elevated temperature (L^Dip^N)AlTe(L^Et^)_2_ equilibrates with dimeric {(L^Dip^N)AlTe(L^Et^)}_2_ in which the chalcogen atoms assume their common role as bridges between the metal centres. These findings demonstrate that (L^Dip^N)AlTe(L^Et^)_2_ comprises the elusive Al=Te double bond in the form of an *N*-heterocyclic carbene-stabilized species.

## Abstract (web summary)

As the structure of bulk aluminium chalcogenides is composed of three dimensional networks their molecular congeners exhibit a strong tendency to oligomerize. Here, the authors describe the isolation of a monotopic aluminium telluride and probe its structural and bonding characteristics.

## Graphical abstract

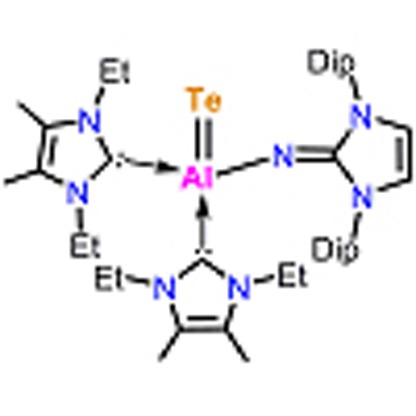

Aluminum is the third most abundant element in the earth's crust surpassed in quantity only by oxygen and silicon and it is most often found among the elements of the group 13 family. The metal is easily oxidized and mostly encountered in the form of aluminum oxides, which are ionic compounds with high lattice energies. The structural diversity of aluminum oxides has led them to be used in a variety of applications in science and technology^1^,^2^,^3^. Among the heavier aluminum chalcogenides, tellurium compounds are explored the least, which is mainly due to this element's low abundance on our planet. Applications of aluminum tellurides in chemical synthesis have barely been reported^4^; however, attention to the heaviest non-radioactive chalcogen has intensified lately because of its use for electronic devices^5^,^6^. Considering the general importance of inorganic aluminum chalcogenides to applied research, it is of prime interest to understand the aggregation processes that create the bulk materials from single atoms and to clarify the nature of the chemical bond between the elements. Theoretical calculations, as well as low-temperature matrix isolation techniques have been used to get an initial insight into the ultrafast bond formations involved in those processes, but these methods have considerable limitations^7^,^8^,^9^. One of the best methods to overcome these limitations is to study well-defined molecular complexes that are stable in the condensed phase at ambient temperatures. Astonishingly, complexes of the general formula R–Al=E (**I**, Fig. 1, R=monoanionic ligand, E=chalcogen) with a double bond between the elements are scarcely found in the literature^10^ and oligomeric compounds (RAlE)_*n*_ (*n*=2, 3, 4, …) dominate the field^11^,^12^,^13^,^14^,^15^.

Interestingly, the double-bonded species could be regarded as a molecular mimic of an elusive intermediate in the processes of aggregation and polymerization that may lead to bulk inorganic aluminum chalcogenides reminiscent of the formation of macromolecular polyolefins from monomolecular vinylic precursors. An apparent explanation for the high reactivity of **I** is the electropositive nature of the aluminum atom, which renders the Al=E moiety a highly polarized Lewis acid–base pair. In consequence, the combination of an electrophilic site at the Al centre and a nucleophilic lone pair located at the chalcogen atom results in self-oligomerization. By the use of uncharged electron pair donor ligands, one can decrease the Lewis acidity of the metal centre to thermodynamically stabilize the Al=E functionality, a technique often complemented by the addition of a Lewis acid that binds to the chalcogen atom. This results in donor–acceptor-stabilized complexes of type **II** (Fig. 1). Further stabilization can be achieved by introduction of an additional Lewis base (**III**, Fig. 1).

This technique has led to the isolation of a monoalumoxane complex of the highly unstable R–Al=O functionality, for which two Lewis bases and one Lewis acid were used to stabilize the Al and O atom, respectively^16^. Though donor–acceptor stabilization is an elaborate and useful synthetic tool to generate complexes with considerable multiple bond character, the steric and electronic stabilization at the terminal chalcogen atom of R–Al=E species significantly limits the investigation of their reactivity. In this work, we describe the monotopic aluminum telluride (L^Dip^N)AlTe(L^Et^)_2_ (**3**, L^Dip^=1,3-(2,6-diisopropylphenyl)-imidazolin-2-imine, L^Et^=1,3-diethyl-4,5-dimethyl-imidazolin-2-ylidene), an electron-precise aluminum chalcogenide that bears a terminal atom of the group 16 family. The unique bonding situation of **3** results in the shortest aluminum–tellurium distance and the highest bond order reported for this type of molecular complex. Furthermore, **3** dimerizes to {(L^Dip^N)AlTe(L^Et^)}_2_ (**5**) at elevated temperature. We interpret these results in terms of an Al=Te double bond to be present in **3**.

## Results

### Synthesis of the monotopic aluminum telluride 3

The synthesis of the aluminum telluride **3** starts out with the previously reported^17^,^18^ dimeric aluminum dihydride **1** (Fig. 2). The introduction of tellurium to the system was accomplished by addition of two equivalents of the convenient tellurium transfer reagent ^n^Bu_3_PTe (^n^Bu=*n*-butyl) to produce the aluminum ditelluride **2** via a dehydrogenative three-electron redox process in which the chalcogen is reduced from the oxidation state of +2 to −1. An X-ray study on **2** revealed that the four-membered Al_2_N_2_ ring is decorated with a bridging ditelluride group. Though we could not obtain crystals of sufficient quality to allow for the discussion of structural parameters, the connectivity as in the structural formulation **2** was verified (Supplementary Fig. 16).

The Al–Te–Te–Al structural motif is without precedence in the literature. Notably, it resembles the Al–Te–Al moiety reported for a pyrazolato aluminum telluride, wherein one tellurium atom bridges the aluminum centres^19^.

Bicyclic systems, such as the Al_2_N_2_Te_2_ moiety in **2**, obviously possess high ring strain because the geometry imposes internal bond angles significantly <109.5°, which aggravates typical tetrahedral coordination. Thus, it seemed reasonable that the cycle might be prone to the cleavage by nucleophilic attack at the metal centres to furnish a monotopic aluminum–tellurium complex. We chose *N*-heterocyclic carbene (NHC) as a nucleophile because NHCs are strong σ-electron pair donors that have proven viable for the taming of numerous elusive main group element compounds on a molecular level^20^,^21^,^22^,^23^,^24^. Indeed, the reaction of the ditelluride **2** with two equivalents of L^Et^ yielded the monotopic telluride **3** (Fig. 2). During this dehydrogenative redox process, the tellurium atom is further reduced to the −2 oxidation state with concomitant formation of dihydrogenated NHC (L^Et^(H_2_), hydrogenated at the formerly carbenoid atom). The abstraction of hydrogen from iminoboranes using NHC to produce NHC(H_2_) had been previously investigated^25^.

The single-crystal X-ray analysis unambiguously shows that the aluminum centre in **3** is bonded to one imino group, two NHC ligands and a terminal tellurium atom (Fig. 3a). The aluminum centre is coordinated in a distorted tetrahedral manner with the chalcogen atom tilted away from the bulkier diisopropylphenyl (Dip) moieties of the imino group towards one of the sterically less hindered carbene ligands. The Al–Te bond length was determined to be 2.5130(14) Å, which is decreased in comparison with the typical range found for molecular aluminum tellurides with four-coordinate aluminum atoms (2.562–2.750 Å)^11^,^15^,^26^,^27^,^28^. Remarkably, the Al–Te distance in **3** is even shorter than the 2.549(1) Å found for the ditopic Te{Al(CH(SiMe_3_)_2_)_2_}_2_ with three-coordinate aluminum centres that marked the hitherto shortest AlTe contact reported for a molecular complex^29^. The terminal position of the tellurium atom in **3** is a unique finding because chalcogens commonly function as bridging ligands in aluminum compounds or are coordinated to another Lewis acid. The closest distance between an aluminum and a tellurium centre in **3** of molecules adjacent in the crystal lattice amounts to 7.31 Å.

### Theoretical study of monotopic aluminum telluride 3

We calculated a Wiberg Bond Index (WBI) of 1.20 for the AlTe bond in **3**, which indicates significant double-bond character, particularly, if the strong polarization along the AlTe vector is taken into account (selected natural population analysis (NPA) charges for **3**: Al=+1.24, Te=−0.95). Notably, it is commonly acknowledged that WBI values have a tendency to underestimate bond orders for heavier main group element bonds. For comparison the WBI_AlTe_ for the ditopic Te{Al(CH(SiMe_3_)_2_)_2_}_2_ (*vide supra*) was calculated using the same theoretical method as for **3** and found to be smaller than one (0.91). This suggests that a trigonal-planar coordination environment of the aluminum centre is not a prerequisite for relevant AlTe double-bond character. Moreover, the WBI_AlTe_ of 1.20 for **3** is in line with that reported for the highly polarized Ge=O double bond in a germanone (WBI_GeO_=1.252) and the Si=O double bond in a sila-urea (WBI_SiO_=1.29), respectively^30^,^31^. Remarkably, the two latter compounds bear trigonal-planar-coordinate metal centres. The molecular orbital analysis of **3** reveals the highest occupied molecular orbital (HOMO) to comprise a lone pair with π-symmetry that is primarily located at the tellurium atom (Fig. 4). The HOMO−1 shows a π-symmetric orbital lobe expanding between the aluminum and the tellurium centre that hints towards an interaction of higher bond order between these atoms. An expected AlTe σ-bond is observed in the HOMO−3 (Supplementary Fig. 21). Antibonding π-interaction among the phenyl rings of the Dip groups is found for the lowest unoccupied molecular orbital (LUMO), which suggests that electron donation to **3** would preferably start out at the ligand system rather than the metal site.

Natural Resonance Theory (NRT) investigation of **3** reveals a major contribution (77%) of resonance structures that feature an Al=Te double bond (that is, **3**) and only a minor share (23%) of zwitterionic species with an Al–Te single bond as **3A** or **3B** (Fig. 5). This result is in accordance with a recent NRT study on NHC-stabilized sila-acylium ions that are isoelectronic to the aluminum telluride **3** (ref. 32). Interestingly, though solid batches of **3** can be stored in an inert atmosphere for weeks the compound decomposes in deuterated tetrahydrofurane solution within few days to yield the protonated iminato ligand (L^Dip^NH, Supplementary Fig. 6).

### Thermal dimerization of 3 to tellurium-bridged 5

When we heated a solution of **3** in benzene dimerization to **5** occurred (Fig. 2) in which the four-membered Al_2_Te_2_ cycle shows the chalcogen-bridged connectivity pattern common for complexes of aluminum with group 16 ligand atoms^14^,^15^,^26^,^27^,^28^. Monitoring the process with NMR spectroscopy in C_6_D_6_ solution revealed that the system equilibrates at 80 °C in about 24 h with the dimer (**5**) and the monomer (**3**) coexisting in an approximate ratio of 1:1 (Supplementary Figs 11 and 12). After **5** has been isolated and stripped from solvent, it decomposes at ambient temperature within hours to a considerable degree (Supplementary Figs 13 and 14). Presumably, the stability of **5** depends on the presence of **3** and free NHC in solution. In comparison with monotopic **3**, the Al–Te distances in **5** are expanded and amount to 2.6143(14) and 2.6211(15) Å (Fig. 3b). This increase in bond length corresponds to the significantly lower calculated bond index of the AlTe interaction in **5** (WBI_AlTe_=0.75) and clearly implies a polarized aluminum tellurium single bond (selected NPA charges for **5**: Al=+1.21, Te=−0.79). Thus, the immense impact of the chalcogen atom's coordination mode (bridging versus terminal) on the type of bonding to the aluminum atom (single versus double bond) is underlined.

DFT analysis of the possible reaction mechanism for the transformation of **3** into **5** revealed intermediate complex **4** (Fig. 2) with a three-coordinate aluminum centre. The formation of **4** from **3** is a barrierless process that is usual for NHC-coordinated main group element structures^32^ and underlines the donor–acceptor-bonding mode^33^ in NHC–Al bonds. The dissociated pair of complex **4** and free L^Et^ is only 15.0 kcal mol^−1^ higher in Gibbs free energy than its precursor **3**, while a comparably low energy gain of 2.9 kcal mol^−1^ is obtained in the overall process of forming the dimer **5** and free L^Et^ from **3** (Supplementary Fig. 22). This small difference in the thermodynamic stabilities of **3** and **5** allows for assuming dynamic equilibrium between the two compounds that explains that **3** does not fully convert into **5** on heating.

## Discussion

**Figure 1:**
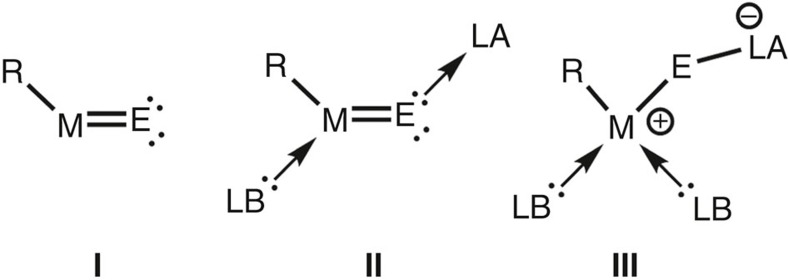
General types of molecular group 13 metal chalcogenides with double-bond character. The parent complex (**I**), as well as the donor–acceptor-stabilized species **II** and **III** (R=anionic ligand, LA=Lewis acid, LB=Lewis base).

**Figure 2:**
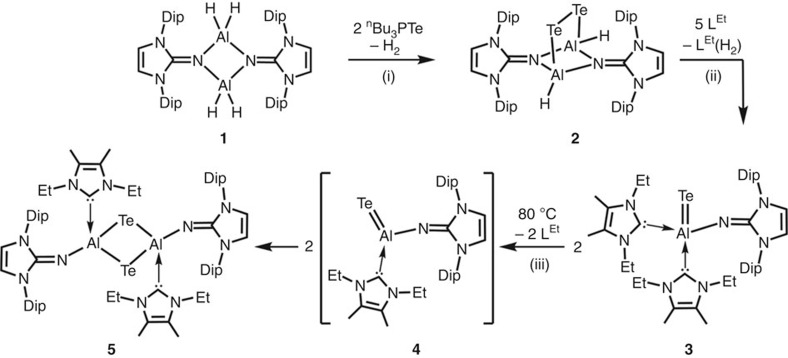
Synthetic overview for this work. Conversion of dimeric aluminum dihydride **1** via ditelluride **2** to monotopic aluminum telluride **3** and its dimerization via intermediate **4** to tellurium-bridged **5** (Dip=2,6-diisopropylphenyl, L^Et^=1,3-diethyl-4,5-dimethyl-imidazolin-2-ylidene). (i) Toluene, rt, 48 h. (ii) Benzene, rt, 48 h. (iii) Benzene, 80 °C, 22 h; rt=ambient temperature.

**Figure 3:**
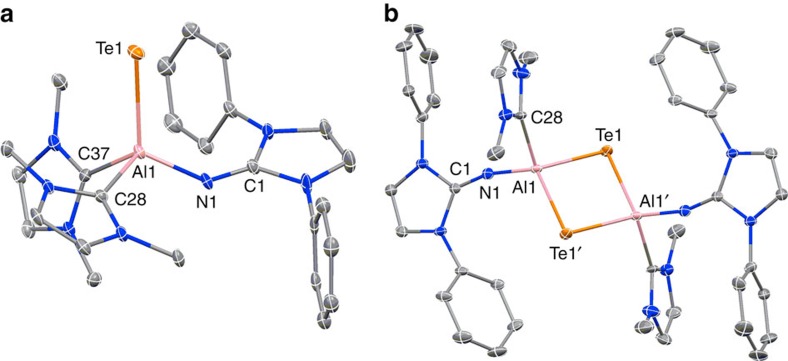
Ellipsoid plots derived from single-crystal X-ray diffraction data. Molecular structures of **3** (**a**) and **5** (**b**) in the solid state. Hydrogen atoms, isopropyl groups and non-N-bonded methyl groups have been omitted for reasons of clarity. Thermal ellipsoids are at the 30% probability level. Selected bond lengths (Å) and bond angles (°): **3**: Al1–Te1=2.5130(14), Al1–N1=1.804(4), Al1–C28=2.084(5), Al1–C37=2.077(5), N1–C1=1.259(6); Te1–Al1–N1=118.34(14), Te1–Al1–C28=102.65(13), Te1–Al1–C37=119.08(14), Al1–N1–C1=139.8(4). **5**: Al1–Te1=2.6211(15), Al1–Te1′=2.6143(14), Al1–N1=1.765(5), Al1–C28=2.086(5), N1–C1=1.253(7); Al1–Te1–Al1′=80.86(5), Te1–Al1–Te1′=99.14(5), N1–Al1–Te1=115.83(15), N1–Al1–Te1′=121.13(16), Al1–N1–C1=164.3(4).

**Figure 4:**
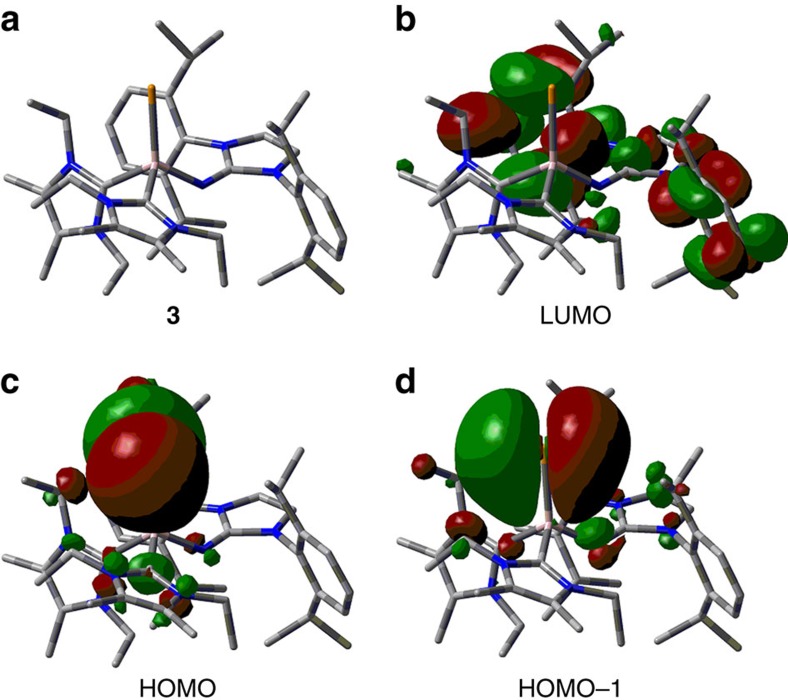
Molecular orbital analysis. Optimized structure and selected molecular orbitals of **3**, the LUMO, HOMO and HOMO–1 ((**a**–**d**) stick models). Te, yellow; Al, pink; N, blue; C, grey. Hydrogen atoms have been omitted for clarity.

**Figure 5:**
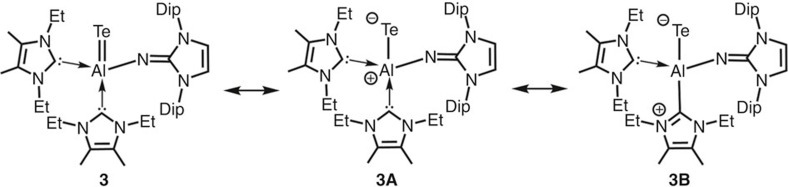
NRT analysis. Selected resonance structures for the aluminum telluride **3**.

As a consequence of the terminal position of the tellurium atom in **3**, the AlTe bond order is markedly increased with respect to complexes in which the chalcogen atom assumes a bridging position. Backed by the results of our theoretical study (WBI, NPA and NRT), we presume that **3** represents the elusive R–Al=Te species (*vide supra*) in the form of an NHC-stabilized congener. Though the aluminum centre in **3** is four-coordinate, the WBI_AlTe_ is comparable to those of polarized metal chalcogen double bonds. Upon thermal dimerization of **3** into **5**, the WBI_AlTe_ of the dimer is vastly decreased, which again underlines the importance of a terminal chalcogen atom for a bond order larger than one. Consequently, an Al=Te double bond is unlikely to be found for complexes following the approach of donor–acceptor stabilization (type **II**, **III**; Fig. 1). The calculated intermediate in the process of dimerization, that is, **4**, which bears a three-coordinate aluminum centre exhibits a higher WBI_AlTe_ than **3** (Supplementary Table 1). There is no experimental proof for its existence, but it is conceivable that the isolation of a more stable congener to **4** may be accomplished by the use of bulkier NHC ligands. However, our conversions of the ditelluride **2** with imidazolin-2-ylidenes more sterically congested than L^Et^ did not yield respective analogues to neither **3** nor **4** or **5**.

The isolation of the monotopic aluminum telluride **3** markedly contributes to the field of aluminum element multiple bonds that are surprisingly unexplored to date. The thermal transformation of **3** into dimeric **5** may serve as a simplified model on the molecular level that could contribute to the elucidation of the complex process of how isolated atoms aggregate to three-dimensional networks in bulk aluminum chalcogenides.

## Methods

### Experimental

All experiments and manipulations were carried out under dry oxygen-free nitrogen using a Schlenk Line or in an MBraun Glove Box Workstation containing an atmosphere of purified nitrogen. Glass junctions were coated with the polytetrafluoroethylene (PTFE)-based grease Merkel Triboflon III. Solvents were dried by standard methods and freshly distilled before the use. NMR spectra were recorded on a Bruker Avance 400 or an Avance III 500 spectrometer. Chemical shift values (*δ*) are given in parts per million and are referenced to (residual) solvent signals (^1^H), and ^13^C{^1^H} NMR. s=singlet, br=broadened, d=doublet, t=triplet, sept=septet, n.o.=not observed, n.r.=not resolved. {L^Dip^NAlH_2_}_2_ (**1**), ^n^Bu_3_PTe and L^Et^ were synthesized according to the published procedures^17^,^34^,^35^. Reagents purchased from commercial sources were used as received if not stated otherwise. High-resolution mass spectrometry (HRMS) was performed on a Thermo Fisher Scientific LTQ Orbitrap XL machine using an atmospheric-pressure chemical ionization (APCI) or an electrospray ionization (ESI) ion source and positive detection mode. Elemental analyses (via combustion) were carried out by the microanalytical laboratory of the Institut für Chemie, Technische Universität Berlin. For depiction of selected NMR spectra see Supplementary Figs 1–14. For NMR spectroscopic characterization of L^Et^(H_2_) see Supplementary Fig. 15 and Supplementary Methods. For details to X-ray analyses see Supplementary Tables 6–8 and Supplementary Methods.

### Computational

Geometry optimizations were carried out at B97-D/6–31G(d)[Al: cc-pVTZ, Te: cc-pVTZ-PP] level of theory in all cases^36^,^37^,^38^,^39^. All stationary points were verified via frequency calculations. All analyses (WBI, NPA, NRT and orbital) occurred at the same level. In case of the reaction mechanism calculation, accurate single-point energy calculations at ωB97X-D/cc-pVTZ[Te: cc-pVTZ-PP] level were performed^40^. Thermodynamic corrections were calculated at 353 K to model reaction conditions. Benzene solvent was considered via the polarizable continuum model, in the framework of the solvation model based on density method^41^. All calculations were carried out with the GAUSSIAN 09 program package^42^. For geometry optimized structures of **2**–**5** see Supplementary Figs 17–20 (depiction) and Supplementary Tables 2–5 (cartesian geometry).

### Synthesis and isolation of {L^Dip^NAl(H)Te}_2_ (2)

In a Glove Box Workstation, a 25-ml Schlenk flask equipped with a PTFE-coated magnetic stirrer bar was charged with {L^Dip^NAlH_2_}_2_ (**1**, 898 mg, 1.04 mmol) and ^n^Bu_3_PTe (733 mg, 2.22 mmol). At the Schlenk Line, toluene (9 ml) was added via syringe to the stirring mixture of solids and the reaction was continued for 48 h (note: gradual evolution of hydrogen gas). A blue suspension was obtained that was diluted with hexane (13 ml). The solid was collected on a frit and the filter cake washed thoroughly with hexane (12 ml) before it was compressed using a pressure gradient. The product was dried in vacuum for 3 h after which 906 mg of **2** in the form of a blue powder was obtained from the frit (0.81 mmol, 78%).

Crystals for X-ray diffraction analysis formed after storing a dilute solution of **2** in toluene/hexane (that is, the combined filtrates from the work-up procedure) at 0 °C for 2 weeks or by gas-phase diffusion exchange of solvent between a solution of **2** in fluorobenzene and a reservoir of hexane for a period of 1 week. Crystals retrieved from either batch analysed with insufficient data quality for the discussion of structural parameters see Supplementary Data 1.

^1^H NMR (500.1 MHz, C_6_D_6_): *δ*=7.23 (t, *J*_HH_=8 Hz, 4 H, C_6_*H*_3_-4), 7.08 (d, *J*_HH_=8 Hz, 8 H, C_6_*H*_3_-3,5), 5.88 (s, 4 H, NC*H*), 3.14 (n.r., 8 H, C*H*(CH_3_)_2_), 2.30 (br, 2 H, Al*H*), 1.47 (d, *J*_HH_=7 Hz, 24 H, CH(C*H*_3_)_2_), 1.06 (d, *J*_HH_=7 Hz, 24 H, CH(C*H*_3_)_2_); ^13^C{^1^H} NMR (125.8 MHz, C_6_D_6_): *δ*=148.8 (N*C*N), 148.4 (C_Aryl_), 133.4 (C_Aryl_), 130.6 (*C*_6_H_3_-3,5), 124.6 (*C*_6_H_3_-4), 117.0 (N*C*H), 29.2 (*C*H(CH_3_)_2_), 25.9 (CH(*C*H_3_)_2_) and 23.7 (CH(*C*H_3_)_2_).

HRMS (APCI), found (%), {calculated} and assigned: *m*/*z*=1118.3701 (17), {1118.3707}, [M]^+^; the analyte was provided as a solution in C_6_D_6_/toluene.

Elemental analysis calculated (%) for C_54_H_74_Al_2_N_6_Te_2_ [1116.39]: C 58.10, H 6.68, N 7.53; found: C 57.47, H 6.70, N 7.47; the analyte was provided wrapped in silver foil.

### Synthesis and isolation of (L^Dip^N)AlTe(L^Et^)_2_ (3)

In a Glove Box Workstation a solution of L^Et^ (148 mg, 0.97 mmol) in 2.0 ml benzene was prepared in a 4 ml SUPELCO Screw Top Vial equipped with a PTFE-coated magnetic stirrer bar. Under vigorous stirring, powderous {L^Dip^NAl(H)Te}_2_ (**2**, 185 mg, 0.17 mmol) was gradually added from a glass tube. The inner walls of the tube were washed with benzene (0.5 ml) to complete the transfer. Stirring was continued for 15 min and a yellow solution had formed. Agitation of the reaction mixture was stopped and the open vial was placed in a glass vessel that contained a reservoir of hexane (22 ml). The vessel was closed to separate the system from the Glove Box Workstation atmosphere for gas-phase diffusion exchange of solvent. After a period of 48 h, the pale yellow supernatant was decanted from the yellow crystals that had formed (the volume of the liquid phase was determined to 3.5 ml). The residual solid was washed by adding hexane (3.5 ml), briefly stirring the suspension and removing the supernatant via syringe along with smaller particles that did not sediment within half a minute. A small amount of residual solvent was evaporated under reduced pressure and the crystal fraction was homogenized with a spatulum before it was dried in vacuum for 1 h. From the vial 217 mg (74%) of **3** in the form of a yellow solid was collected.

The crystal used for the reported X-ray diffraction analysis was obtained by gas-phase diffusion exchange of solvent between a solution of **3** in fluorobenzene and a reservoir of Et_2_O for a period of 1 week, Supplementary Data 2. A crystal that had formed from a saturated solution of **3** in C_6_D_6_ analysed to (**3**)_2_·(C_6_D_6_)_7_ (data not reported). Compound **3** slowly decomposes in THF-*d*_8_ solution at ambient temperature (Supplementary Figs 4 and 6) but it is significantly more stable in C_6_D_6_ solution (Supplementary Figs 7 and 8). Solid batches of **3** were stored in a Glove Box Workstation at ambient temperature for weeks without notable change in the NMR spectroscopic analysis; however, it was observed in some instances that the substance was covered with a thin layer of brown colour. Thus, storage of **3** in an inert atmosphere at low temperature is recommended.

^1^H NMR (400.1 MHz, THF-*d*_8_): *δ*=7.23 (t, *J*_HH_=8 Hz, 2 H, C_6_*H*_3_-4), 7.11 (d, *J*_HH_=8 Hz, 4 H, C_6_*H*_3_-3,5), 6.14 (s, 2 H, NC*H*), 4.67 (m, 4 H, C*H*_2_CH_3_), 3.68 (m, 4 H, C*H*_2_CH_3_), 3.51 (sept, *J*_HH_=7 Hz, 4 H, C*H*(CH_3_)_2_), 2.01 (s, 12 H, CC*H*_3_), 1.29 (d, *J*_HH_=7 Hz, 12 H, CH(C*H*_3_)_2_), 1.13 (d, *J*_HH_=7 Hz, 12 H, CH(C*H*_3_)_2_), 0.74 (t, *J*_HH_=7 Hz, 12 H, CH_2_C*H*_3_); ^1^H NMR (400.1 MHz, C_6_D_6_): *δ*=7.23–7.13 (m, 6 H, C_6_*H*_3_-3,4,5)^a^, 6.11 (s, 2 H, NC*H*), 4.81 (m, 4 H, C*H*_2_CH_3_), 3.87–3.73 (m, 8 H, C*H*_2_CH_3_ and C*H*(CH_3_)_2_), 1.54 (d, *J*_HH_=7 Hz, 12 H, CH(C*H*_3_)_2_), 1.51 (s, 12 H, CC*H*_3_), 1.31 (d, *J*_HH_=7 Hz, 12 H, CH(C*H*_3_)_2_), 0.73 (t, *J*_HH_=7 Hz, 12 H, CH_2_C*H*_3_); ^13^C{^1^H} NMR (125.8 MHz, THF-*d*_8_): *δ*=149.6 (C_Aryl_), 142.5 (N*C*N_imino_), 138.4 (C_Aryl_), 128.2 (*C*_6_H_3_-4), 125.0 (C_Aryl_), 124.0 (*C*_6_H_3_-3,5), 114.9 (N*C*H), 42.4 (*C*H_2_CH_3_)^b^, 29.0 (*C*H(CH_3_)_2_), 25.3 (CH(*C*H_3_)_2_)^c^, 24.4 (CH(*C*H_3_)_2_), 15.2 (CH_2_*C*H_3_), 8.3 (C*C*H_3_), n.o. (N*C*N_carbene_)^d^. a=the resonance superimposes with the residual solvent signal which explains its high intensity in Supplementary Fig. 7; b=the ^1^H^13^C-HSQC NMR experiment verifies that the two signals of the C*H*_2_CH_3_ protons in the ^1^H NMR spectrum correlate to one resonance in the ^13^C{^1^H} NMR analysis produced by the *C*H_2_CH_3_ carbon atoms; c=the resonance superimposes with the signal produced by the NMR solvent but is identified via ^1^H^13^C-HSQC NMR analysis; d=observation of the aluminum-bonded ^13^C atoms is hampered by the quadrupolar momentum of the ^27^Al nucleus.

HRMS (ESI), found (%), {calculated} and assigned: *m*/*z*=864.4519 (100), {864.4487}, [M+H]^+^; 1016.5832 (99), {1016.5800}, [M+L^Et^+H]^+^; the analyte was provided as a solution in fluorobenzene.

Elemental Analysis calculated (%) for C_45_H_68_AlN_7_Te [861.67]: C 62.73, H 7.95, N 11.38; found: C 62.54, H 8.14, N 11.41; the analyte was provided wrapped in silver foil.

### Synthesis and isolation of {L^Dip^NAl(L^Et^)Te}_2_ (5)

In a Glove Box Workstation, an NMR sample tube was charged with (L^Dip^N)AlTe(L^Et^)_2_ (**3**, 72 mg, 0.08 mmol) and benzene (0.9 ml). At the Schlenk Line, the mixture was frozen in liquid nitrogen and the tube was flame-sealed under vacuum. After thawing in a water bath at ambient temperature the reaction vessel was wrapped in aluminum foil (as a safety consideration) and stored in an oven at 80 °C for 22 h. In a Glove Box Workstation, the tube was unsealed and the yellow solution was withdrawn via syringe. The inner glass walls and residual solid were washed with benzene (0.6 ml) and the liquid phases were combined in a 9-ml Snap Cap Vial equipped with a PTFE-coated magnetic stirrer bar. Piercing the polyethylene cap with a steel cannula hexane (4.0 ml) was added dropwise via syringe to the vigorously stirred solution over a period of 8 min resulting in the formation of a pale yellow precipitate. The solid was allowed to sediment for 12 h, and then the supernatant (5.0 ml) was withdrawn via syringe and filtered through a 0.45-μm PTFE filter put between the outlet and the steel cannula. The vacuum-dried precipitate (30 mg) was identified as spectroscopically clean **3** via NMR analysis (THF-*d*_8_). The filtrate was stored in a Snap Cap Vial at −33 °C for 4 days. A crystalline solid had formed (*vide infra*) and the liquid phase (4.8 ml) was removed via syringe. The crystal fraction was dried in vacuum for 30 min to yield an off-white powder. From the glass vessel 24 mg (38%) of product were transferred and verified as spectroscopically clean **5** via NMR analysis (C_6_D_6_).

The crystal used for the X-ray diffraction study of **5** was obtained by slow diffusion of hexane into the filtered (0.45 μm PTFE filter) reaction mixture via the gas phase for a period of 3 days, Supplementary Data 3. Afterwards, the batch (crystals+mother liquor) was stored for an additional 2 days in an enclosed glass vessel (screw-cap vial) during which it recrystallized from smaller to larger entities before a crystal was picked. A crystal retrieved from the work-up process before phase separation (*vide supra*) analysed to **5**(C_6_H_6_)_4_ (data not reported). The procedure was reproduced several times; however, in one case removal of the cold solution from the freezer and brief storage at ambient temperature was necessary to initialize the final crystallization process at low temperature.

After isolation as a vaccum-dried solid, **5** slowly decomposes at ambient temperature. A batch stored in the Glove Box Workstation for 20 h showed significant signs of decomposition upon NMR spectroscopic reanalysis (Supplementary Fig. 13). Suitable elemental analysis was found for a batch that had been briefly washed with cold hexane (2 ml) and stored at −30 °C for 12 days. After that period it was briefly dried in vacuum and submitted for combustion analysis without further delay. NMR spectroscopic analysis (C_6_D_6_) verified that the isolated product did not decompose during the prolonged low-temperature storage episode. In C_6_D_6_ solution **5** is sufficiently stable for the recording of NMR spectra but shows signs of decomposition after few hours at ambient temperature (Supplementary Fig. 14).

^1^H NMR (500.1 MHz, C_6_D_6_): *δ*=7.23 (t, *J*_HH_=8 Hz, 4 H, C_6_*H*_3_-4), 7.11 (d, *J*_HH_=8 Hz, 8 H, C_6_*H*_3_-3,5), 5.93 (s, 4 H, NC*H*), 4.07 (m, 8 H, C*H*_2_CH_3_), 3.42 (sept, *J*_HH_=7 Hz, 8 H, C*H*(CH_3_)_2_), 1.67 (s, 12 H, CC*H*_3_), 1.27–1.20 (m, 60 H, CH(C*H*_3_)_2_ and CH_2_C*H*_3_); ^13^C{^1^H} NMR (125.8 MHz, C_6_D_6_): *δ*=149.0 (*C*_6_H_3_-2,6), 137.9 (*C*_6_H_3_-1), 137.2 (N*C*N_imino_), 128.4 (*C*_6_H_3_-4)^a^, 123.5 (*C*_6_H_3_-3,5), 123.2 (*C*CH_3_), 113.7 (N*C*H), 41.7 (*C*H_2_CH_3_), 28.7 (*C*H(CH_3_)_2_), 24.6 (CH(*C*H_3_)_2_), 24.3 (CH(*C*H_3_)_2_), 17.0 (CH_2_*C*H_3_), 8.0 (C*C*H_3_), n.o. (N*C*N_carbene_)^b^. a=the resonance partially superimposes with the signal produced by the solvent but is assigned via ^1^H^13^C-HSQC NMR experiment; b=observation of the aluminum-bonded ^13^C atoms is hampered by the quadrupolar momentum of the ^27^Al nucleus.

Elemental analysis calculated (%) for C_72_H_104_Al_2_N_10_Te_2_ [1418.86]: C 60.95, H 7.39, N 9.87; found: C 60.86, H 7.55, N 9.81; the analyte was provided wrapped in silver foil.

## Additional information

**Accesion Codes:** The X-ray crystallographic coordinates for structures reported in this Article have been deposited at the Cambridge Crystallographic Data Centre (CCDC), under deposition number CCDC 1415006-1415008. These data can be obtained free of charge from The Cambridge Crystallographic Data Centre via www.ccdc.cam.ac.uk/data_request/cif.

**How to cite this article:** Franz, D. *et al.* A monotopic aluminum telluride with an Al=Te double bond stabilized by *N*-heterocyclic carbenes. *Nat. Commun.* 6:10037 doi: 10.1038/ncomms10037 (2015).

## Supplementary Material

Supplementary Information Supplementary Figures 1-22, Supplementary Tables 1-8, Supplementary Methods and Supplementary References

Supplementary Data 1 Crystallographic Information File for compound 2.

Supplementary Data 2 Crystallographic Information File for compound 3.

Supplementary Data 3 Crystallographic Information File for compound 5.
