# Supplementary material for: A monotopic aluminum telluride with an Al=Te double bond stabilized by N-heterocyclic carbenes
Source: Nat Commun. 2015 Nov 27;6:10037. doi: 10.1038/ncomms10037 (PMC4674676; doi:10.1038/ncomms10037)
Supplement: Supplementary Information — Supplementary Figures 1-22, Supplementary Tables 1-8, Supplementary Methods and Supplementary References [file ncomms10037-s1.pdf]

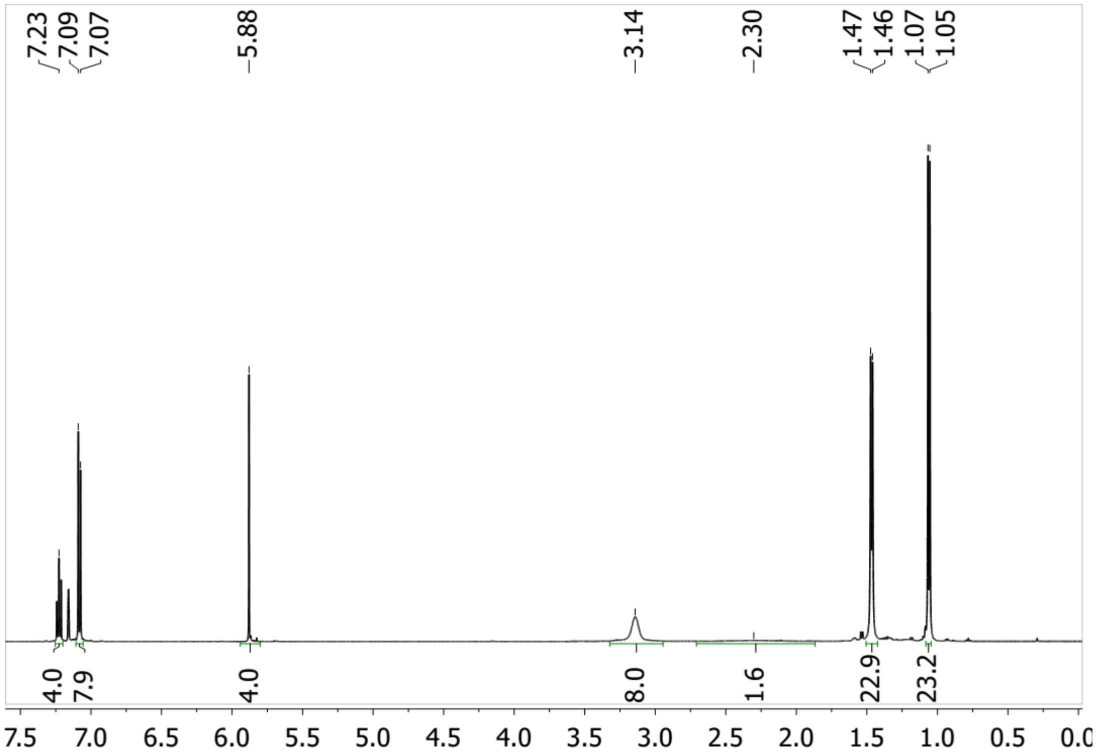

Supplementary Figure 1. ^{1}H NMR spectrum (500.1 MHz) of **2** in C_6D_6.

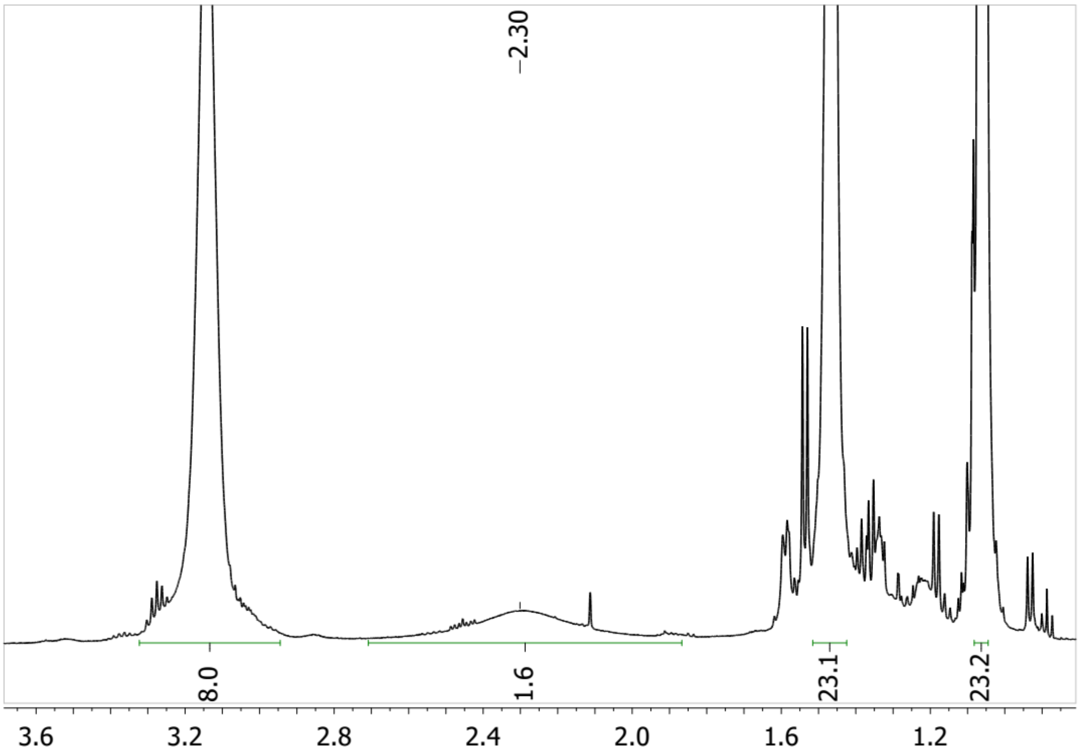

Supplementary Figure 2. Expansion of the high-field region of the spectrum in Supplementary Figure 1 showing the signal produced by the Al*H* hydrogen atoms of **2** at 2.30 ppm.

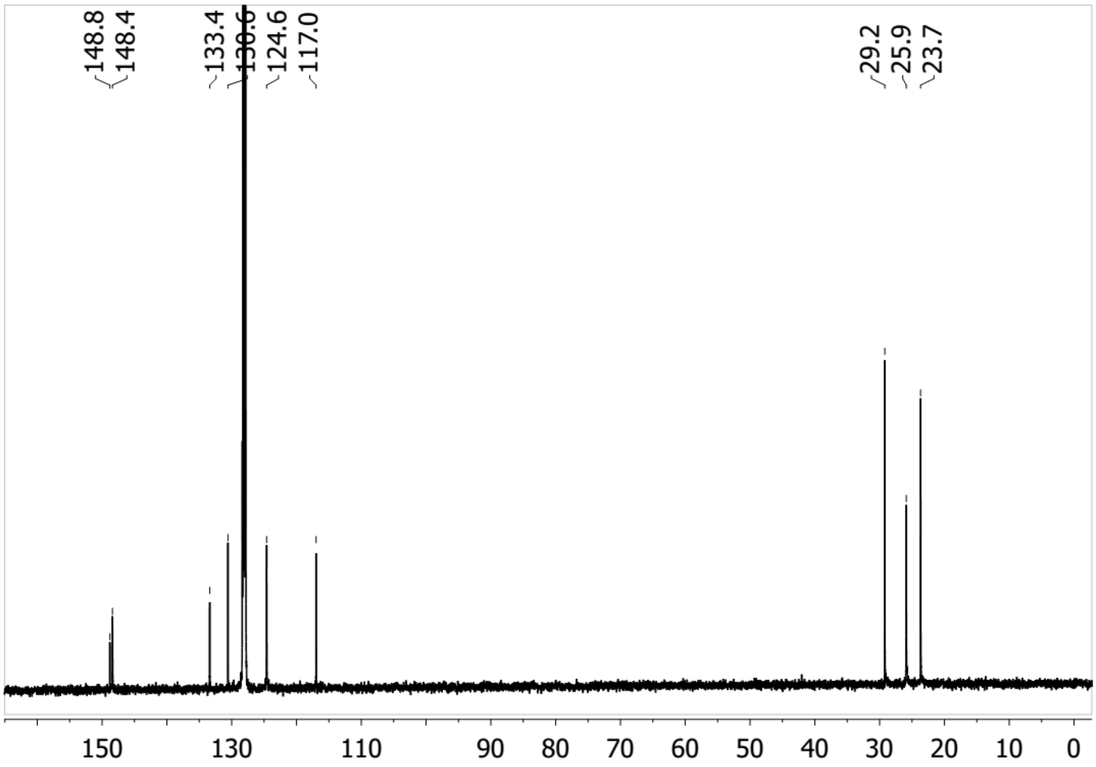

Supplementary Figure 3. $^{13}C\{^{1}H\}$ NMR spectrum (125.8 MHz) of **2** in C_6D_6.

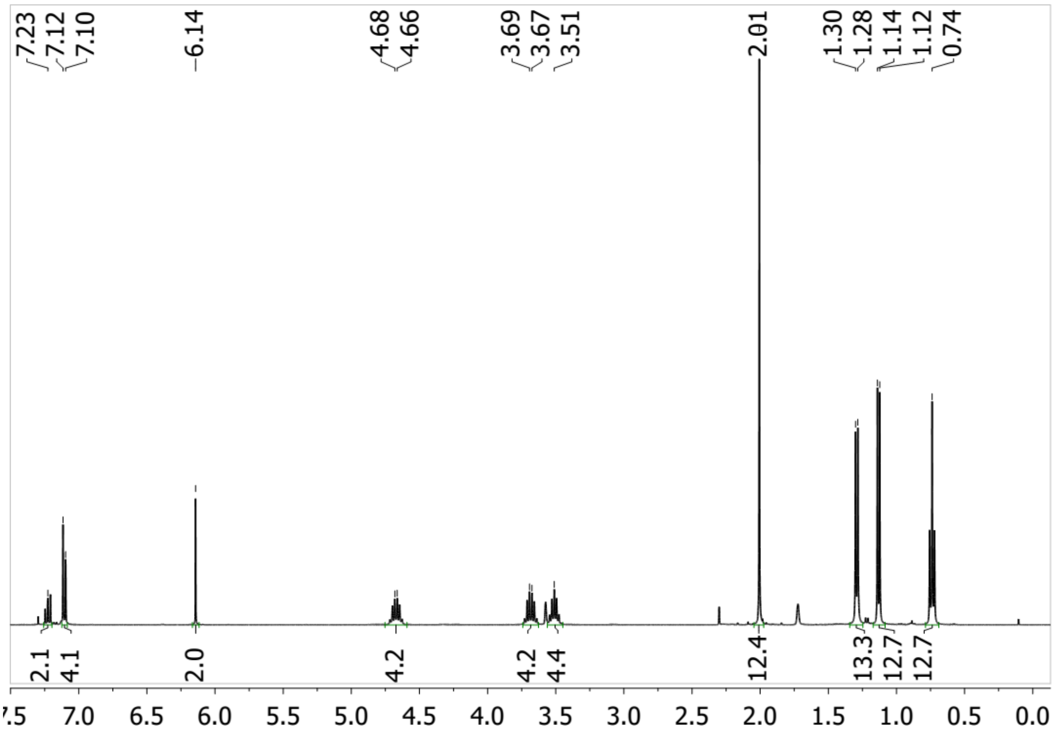

Supplementary Figure 4. ^{1}H NMR spectrum (400.1 MHz) of **3** in THF-d_8.

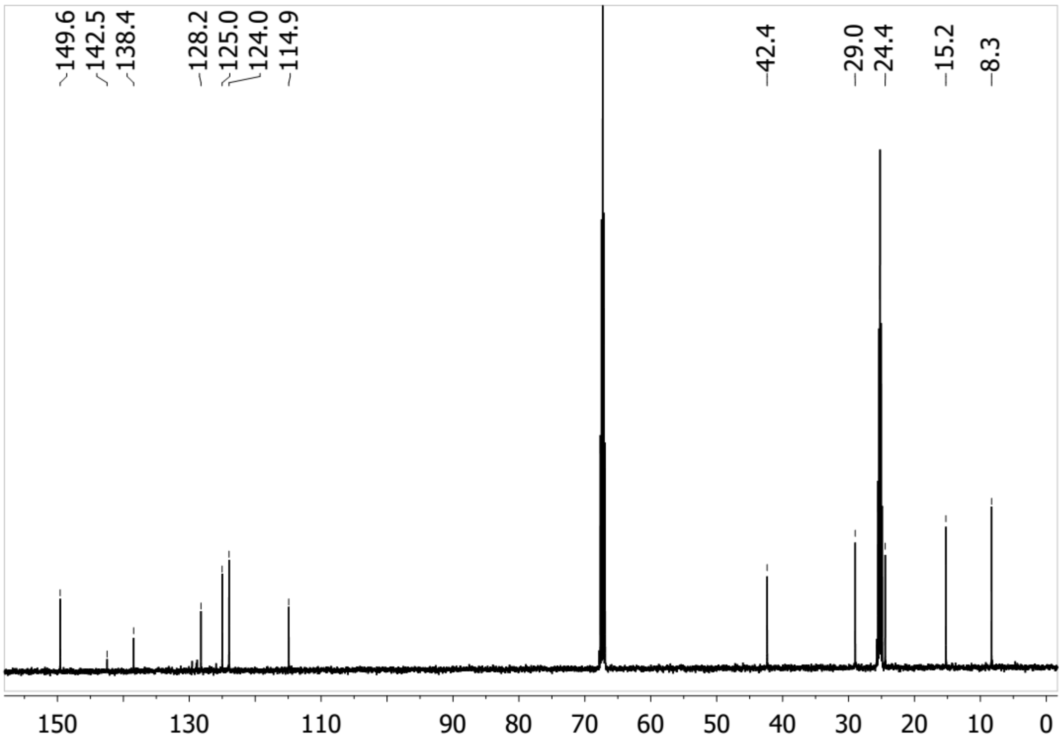

Supplementary Figure 5. $^{13}C\{^1H\}$ NMR spectrum (125.8 MHz) of **3** in THF-d_8.

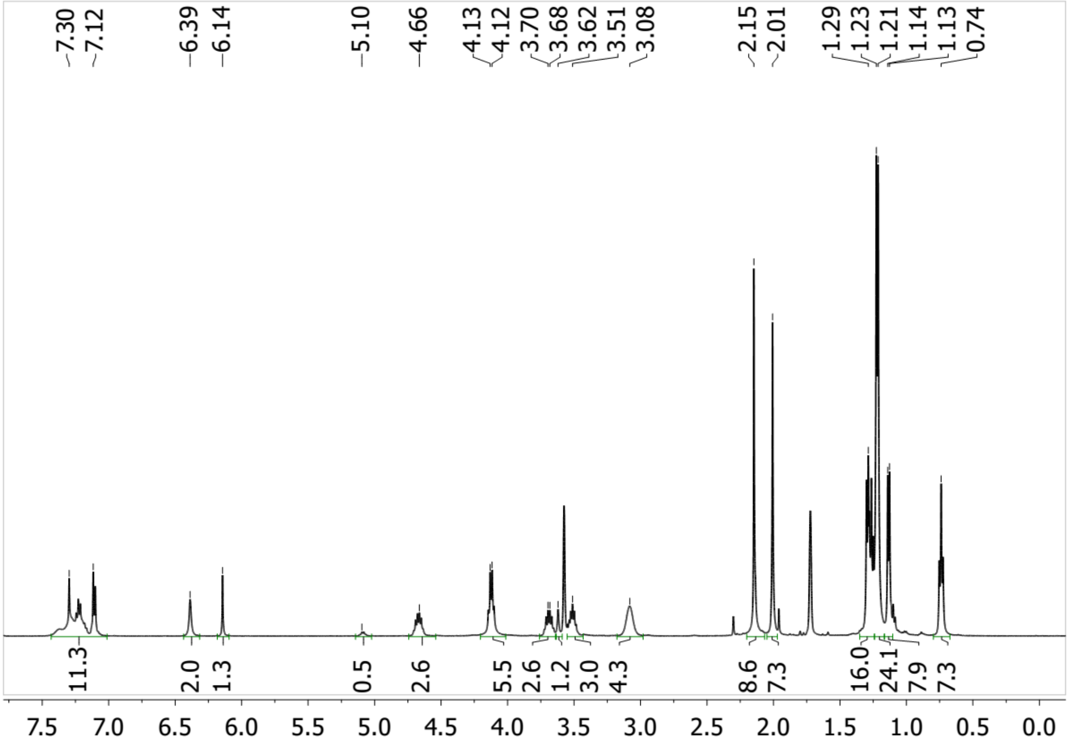

Supplementary Figure 6. ^{1}H NMR spectrum (500.1 MHz) of **3** in THF-d_8 after storage of a flame-sealed sample tube at ambient temperature for 4 days. The spectrum shows L^{Dip}NH and **3** in a 2:1.3 ratio. Significant amounts of an off-white powder had separated from the solution.

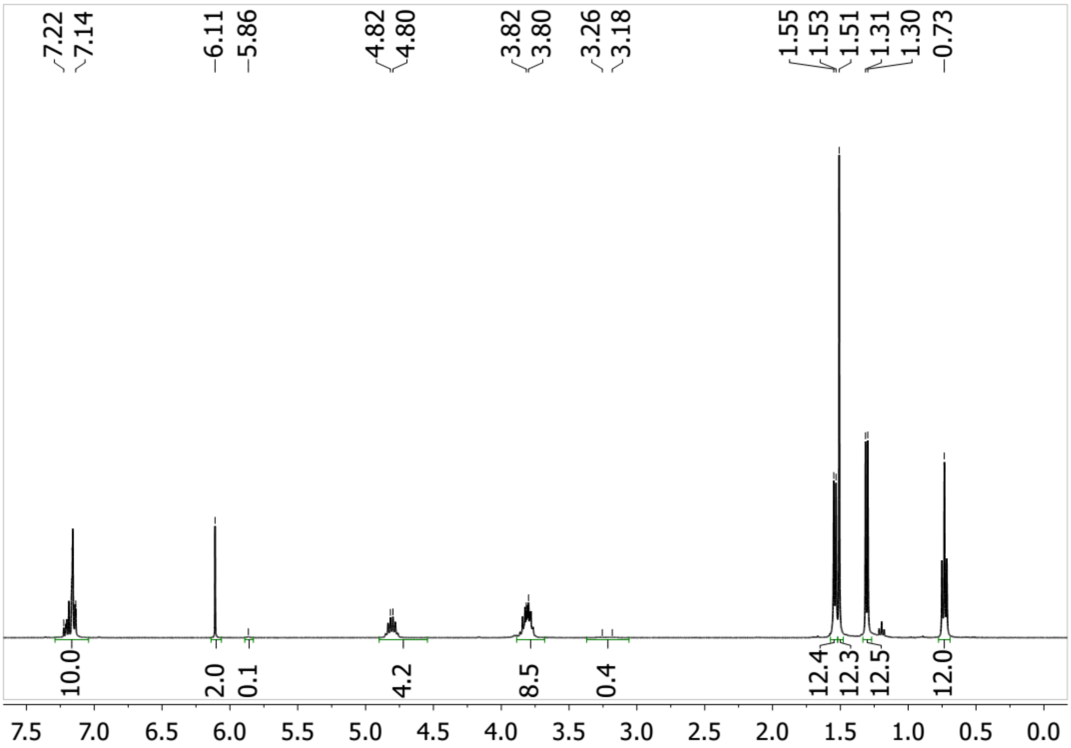

Supplementary Figure 7. ^{1}H NMR spectrum (400.1 MHz) of **3** in C_6D_6. The spectrum shows trace amounts of $L^{Dip}NH$ (selected resonances: 5.86 ppm, s, 0.1 H, NC*H* and 3.22 ppm, br, 0.4 H, C*H*(CH_3)$_2$). Impurities are overestimated due to the poor solubility of **3** in C_6D_6.

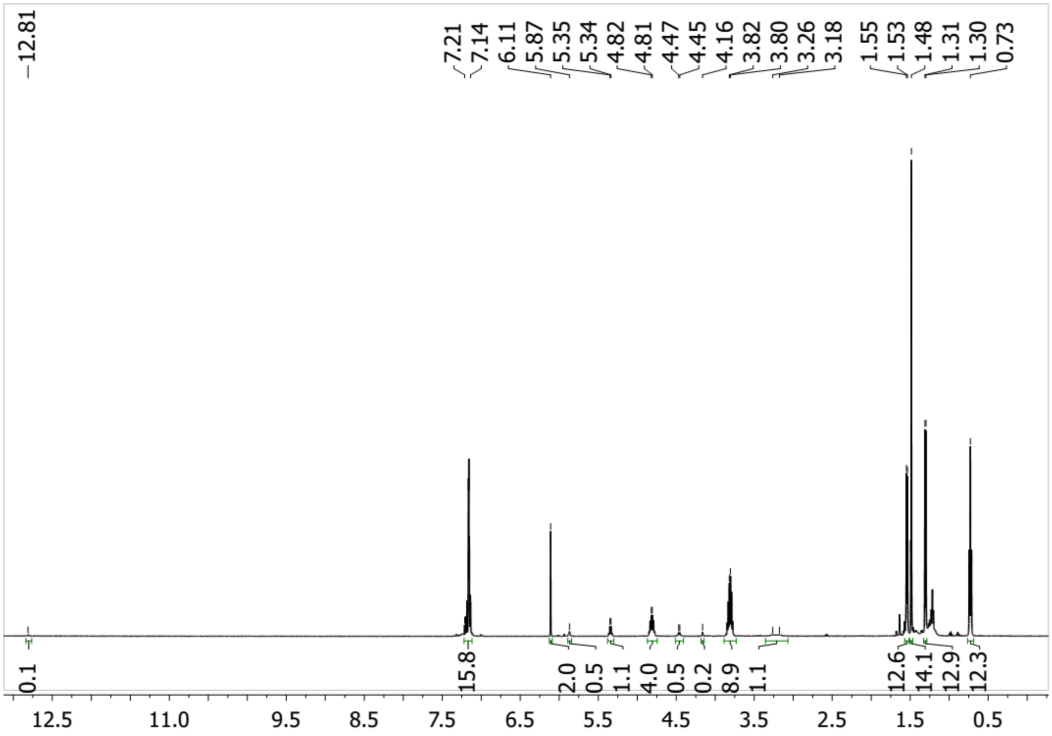

Supplementary Figure 8. ^{1}H NMR spectrum (500.1 MHz) of **3** in C_6D_6 after storage of the flame-sealed sample tube at ambient temperature for 10 days. The spectrum shows $L^{Dip}NH$ (selected resonances: 5.87 ppm, s, 0.5 H, NC*H* and 3.22 ppm, br, 1.1 H, C*H*$(CH_3)_2$) and additional decomposition products. Impurities may be overestimated due to the low solubility of **3** in C_6D_6.

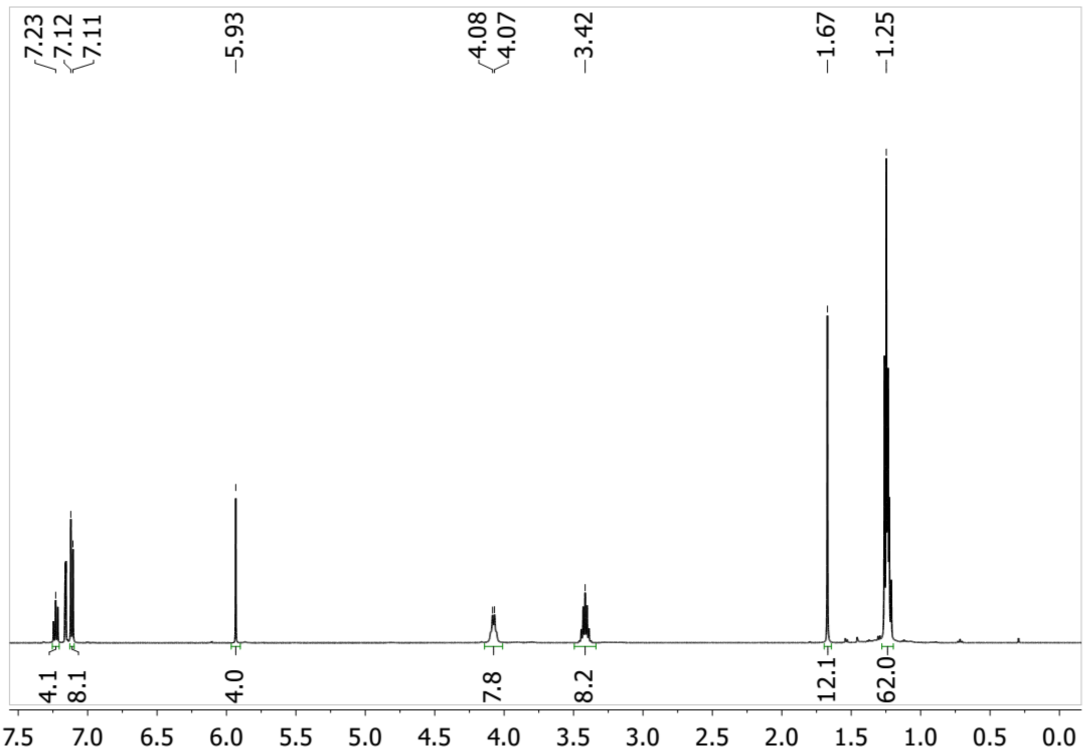

Supplementary Figure 9. ^{1}H NMR spectrum (500.1 MHz) of **5** in C_6D_6.

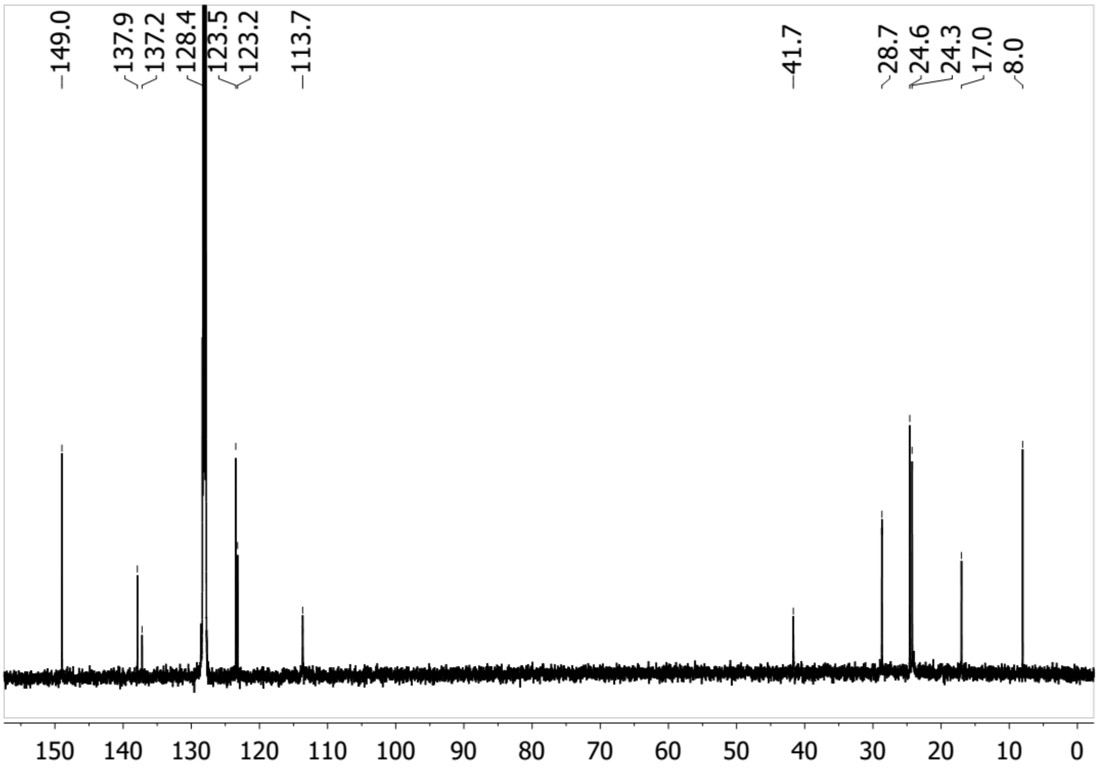

Supplementary Figure 10. $^{13}C\{^1H\}$ NMR spectrum (125.8 MHz) of **5** in C_6D_6.

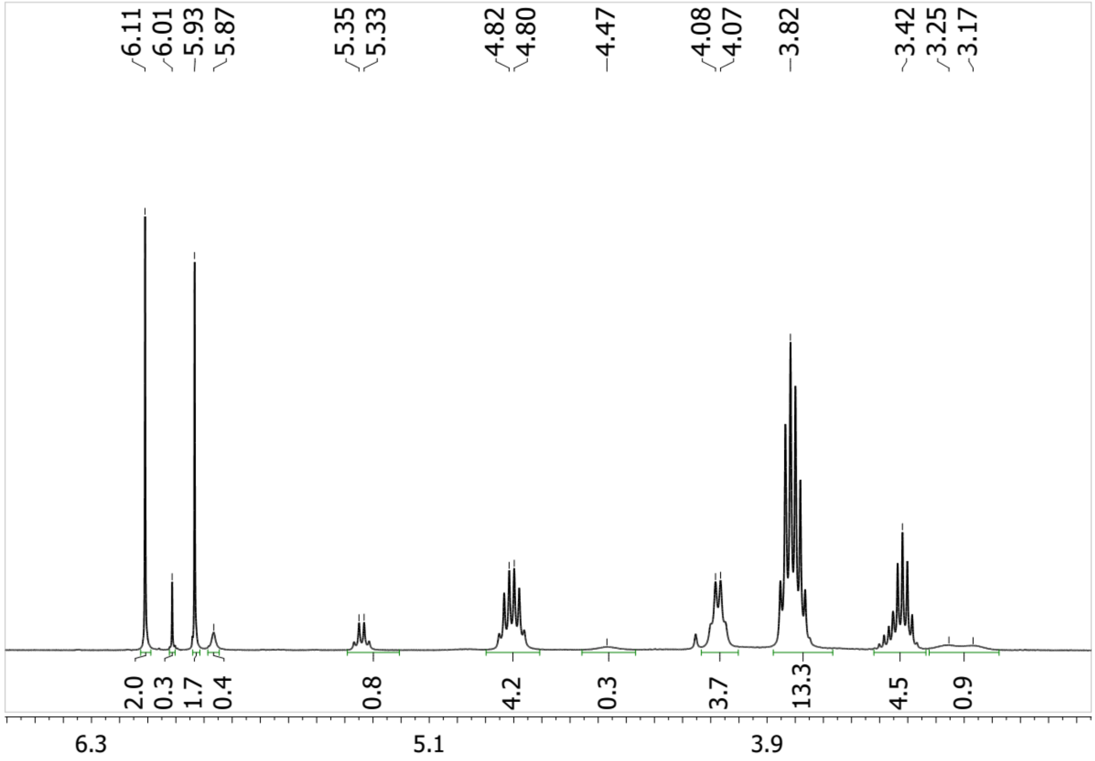

Supplementary Figure 11. Expansion of the ^{1}H NMR spectrum (400.1 MHz) of **3** in C_6D_6 after storage for 6 h at 80 °C (flame-sealed sample tube). From the singlets produced by the respective ligand backbones it is concluded on the presence of **3**, an unknown species, **5** and L^{Dip}NH in a 2.0:0.3:0.9:0.4 ratio in solution.

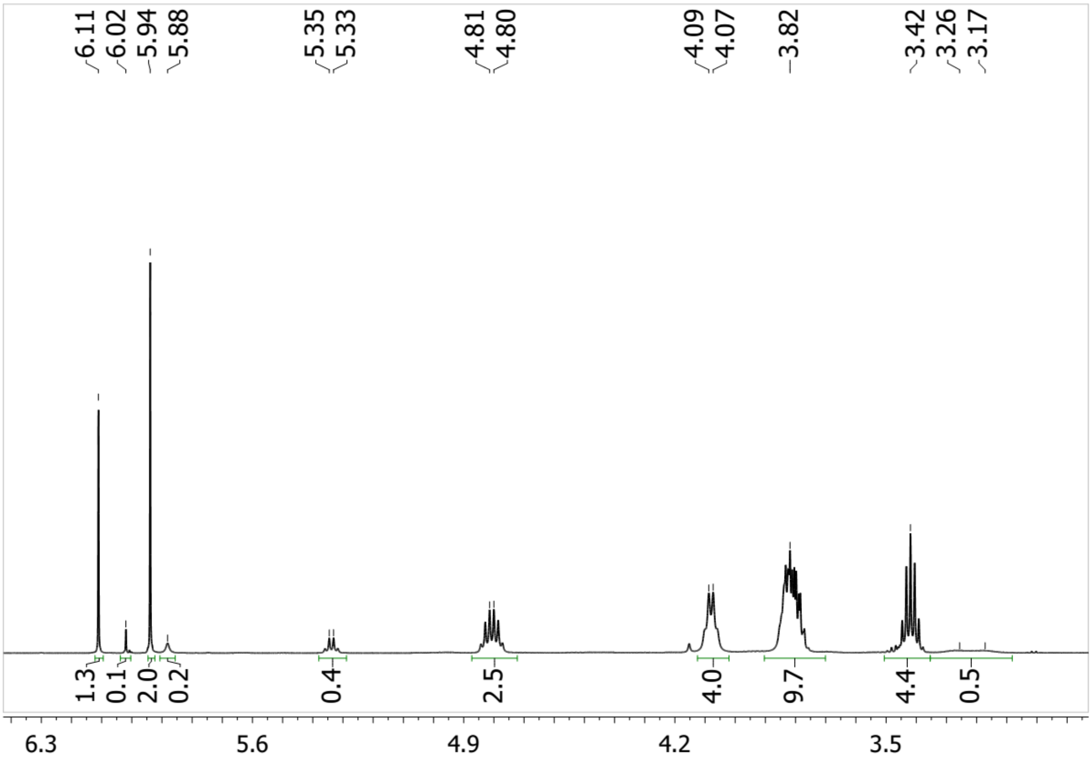

Supplementary Figure 12. Expansion of the ^{1}H NMR spectrum (500.1 MHz) of **3** in C_6D_6 after storage for 26 h at 80 °C (flame-sealed sample tube). From the singlets produced by the respective ligand backbones it is concluded on the presence of **3**, an unknown species, **5** and L^{Dip}NH in a 1.3:0.1:1.0:0.2 ratio in solution.

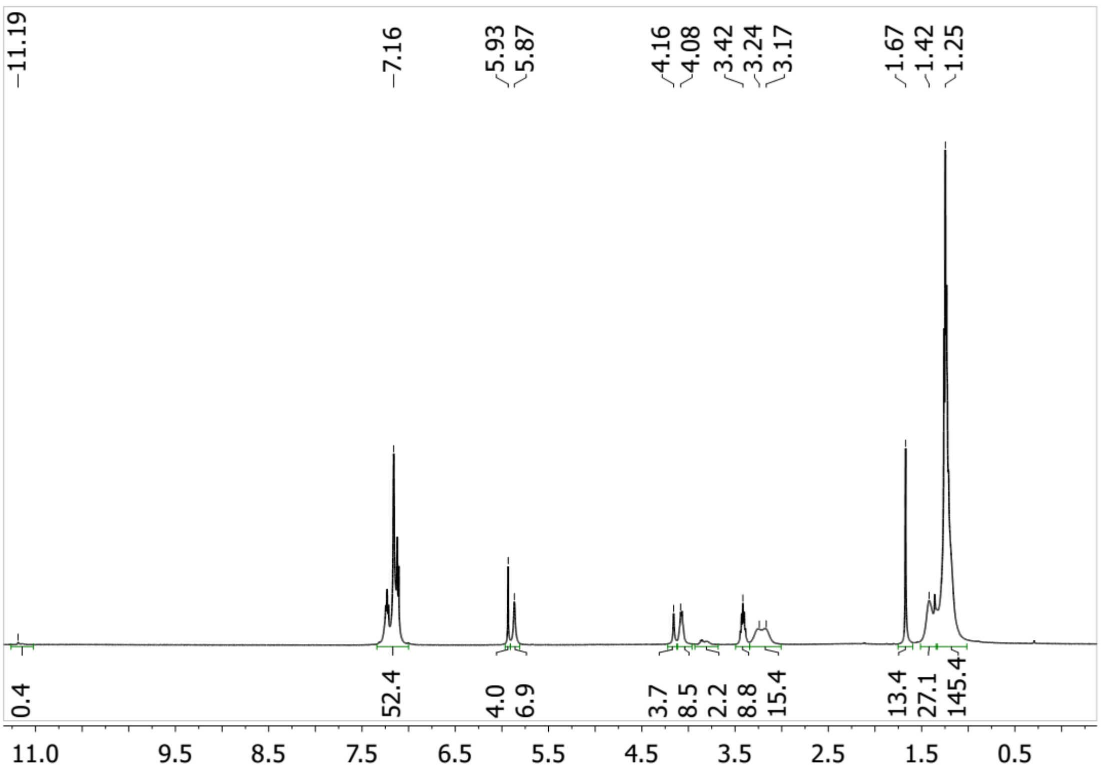

Supplementary Figure 13. ^{1}H NMR spectrum (500.1 MHz) of **5** in C_6D_6 after storage of the isolated solid product in the *Glove Box Workstation* at ambient temperature for 20h. The spectrum shows **5** and $L^{Dip}NH$ in a 1:3.5 ratio, as well as putative trace amounts of $[L^{Et}H]^{+}$.

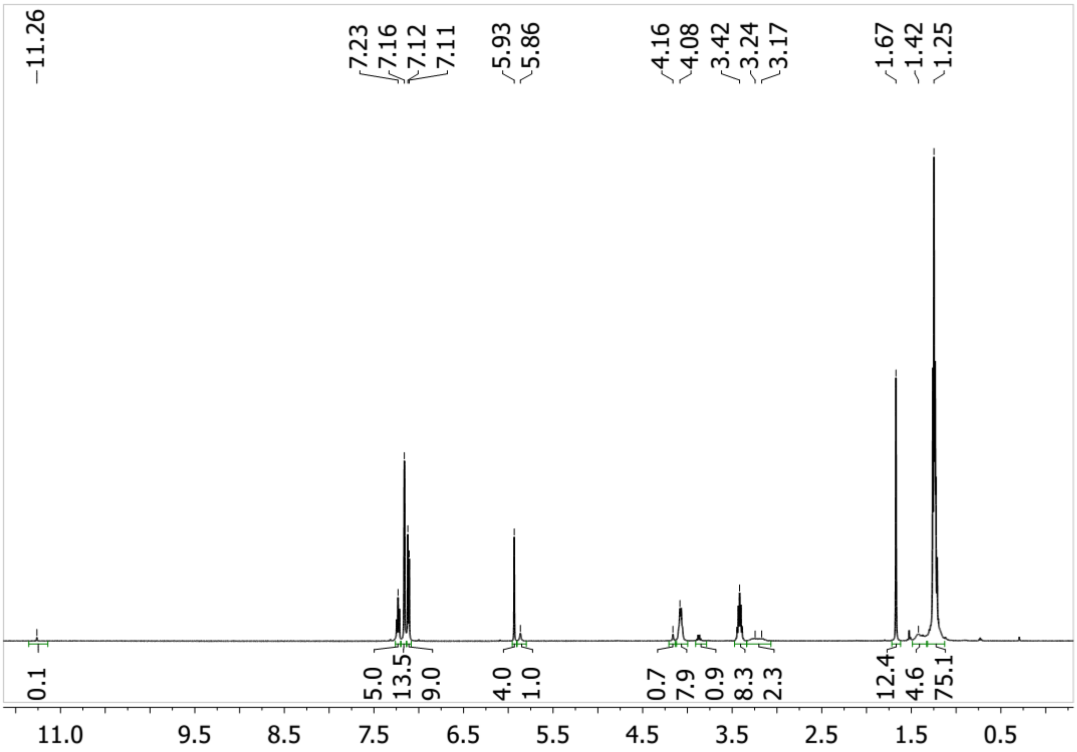

Supplementary Figure 14. ^{1}H NMR spectrum (500.1 MHz) of **5** in C_6D_6 after storage of the rubber cap-sealed sample tube at ambient temperature for 4 h. The spectrum shows **5** and L^{Dip}NH in a 1:0.5 ratio, as well as putative trace amounts of $[L^{Et}H]^{+}$.

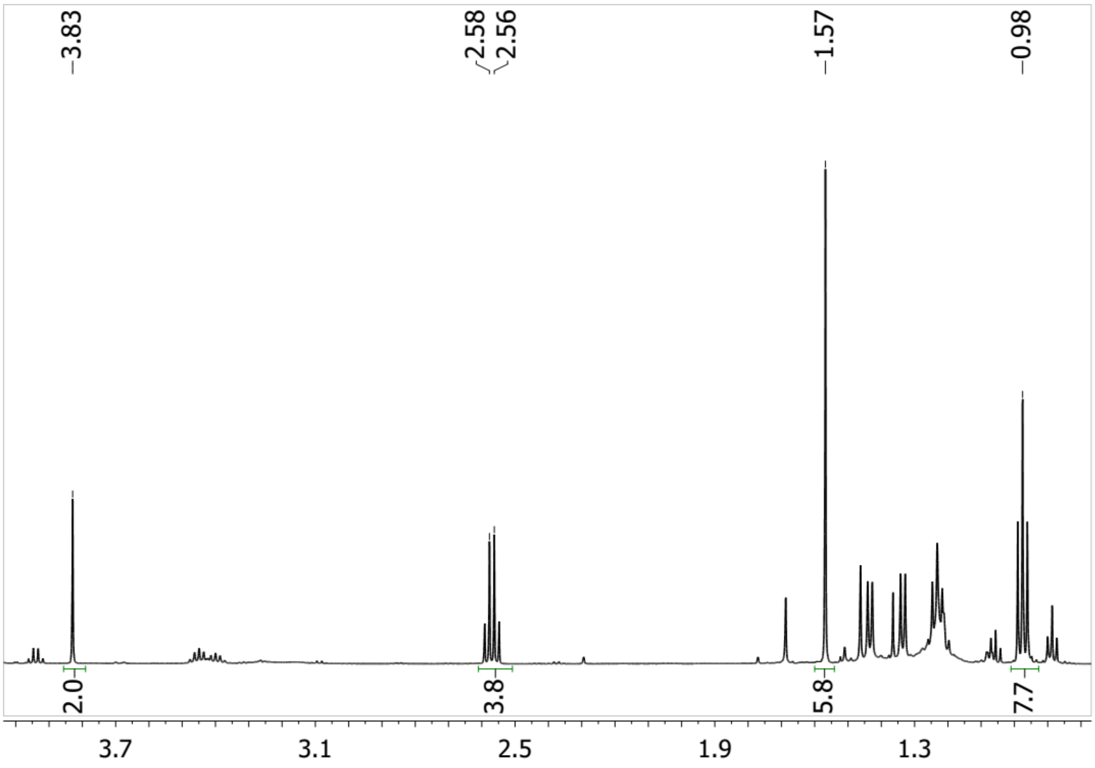

Supplementary Figure 15. ^{1}H NMR spectrum (500.1 MHz) of crude $L^{Et}(H_2)$ in C_6D_6 as derived from the reaction mixture of the synthesis of **3** after separation of **3** (high content of impurities).

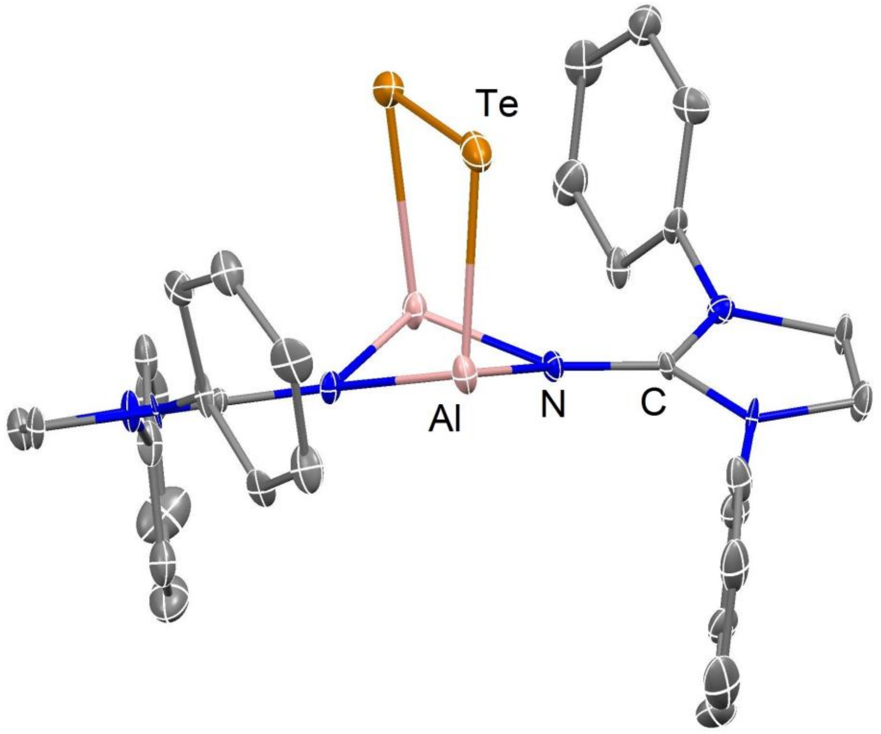

Supplementary Figure 16. Molecular structure of **2** in the solid state derived from single crystal X-ray diffraction analysis. Hydrogen atoms and isopropyl groups have been omitted. Thermal ellipsoids are at the 30% probability level. Structural parameters are not given due to the insufficient quality of the data (cf. Supplementary Table 6).

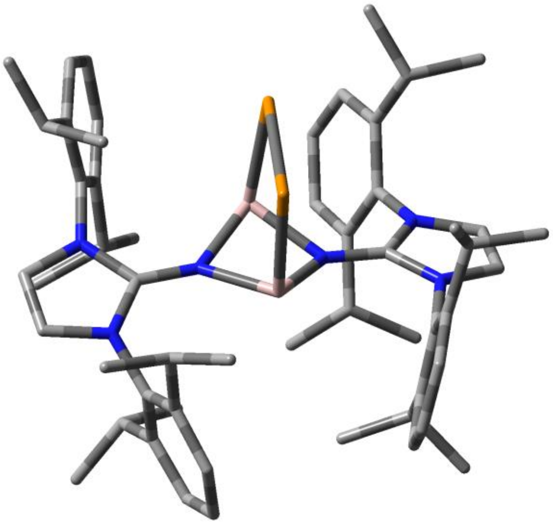

Supplementary Figure 17. Calculated molecular structure of **2** at B97-D/6-31G(d)[Al: cc-pVTZ, Te: cc-pVTZ-PP] level of theory (stick model). Te: yellow, Al: pink, N: blue, C: grey. Hydrogen atoms are omitted for clarity.

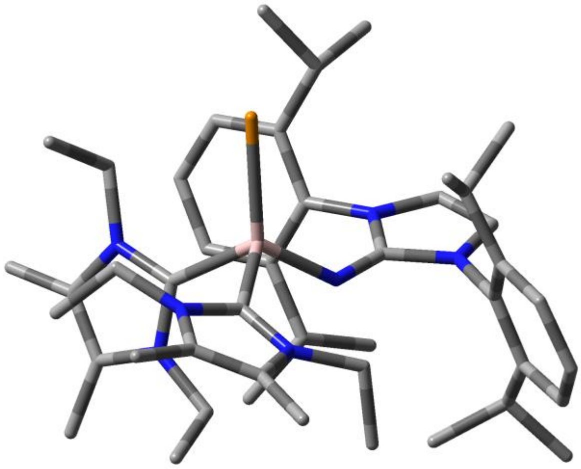

Supplementary Figure 18. Calculated molecular structure of **3** at B97-D/6-31G(d)[Al: cc-pVTZ, Te: cc-pVTZ-PP] level of theory (stick model). Te: yellow, Al: pink, N: blue, C: grey. Hydrogen atoms are omitted for clarity.

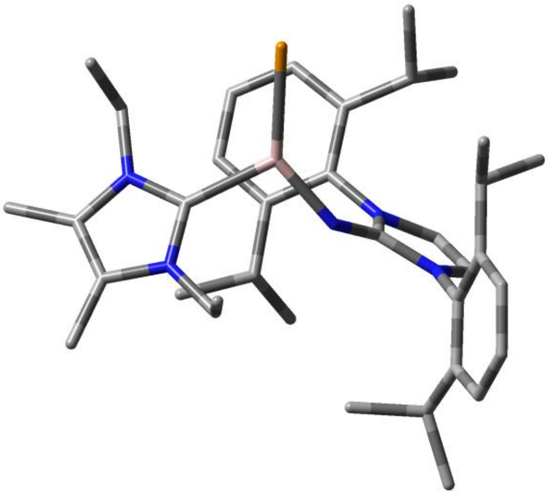

Supplementary Figure 19. Calculated molecular structure of **4** at B97-D/6-31G(d)[Al: cc-pVTZ, Te: cc-pVTZ-PP] level of theory (stick model). Te: yellow, Al: pink, N: blue, C: grey. Hydrogen atoms are omitted for clarity.

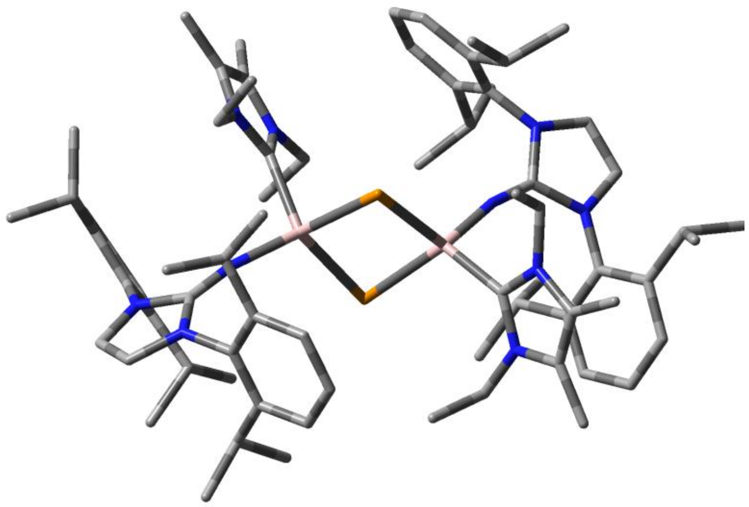

Supplementary Figure 20. Calculated molecular structure of **5** at B97-D/6-31G(d)[Al: cc-pVTZ, Te: cc-pVTZ-PP] level of theory (stick model). Te: yellow, Al: pink, N: blue, C: grey. Hydrogen atoms are omitted for clarity.

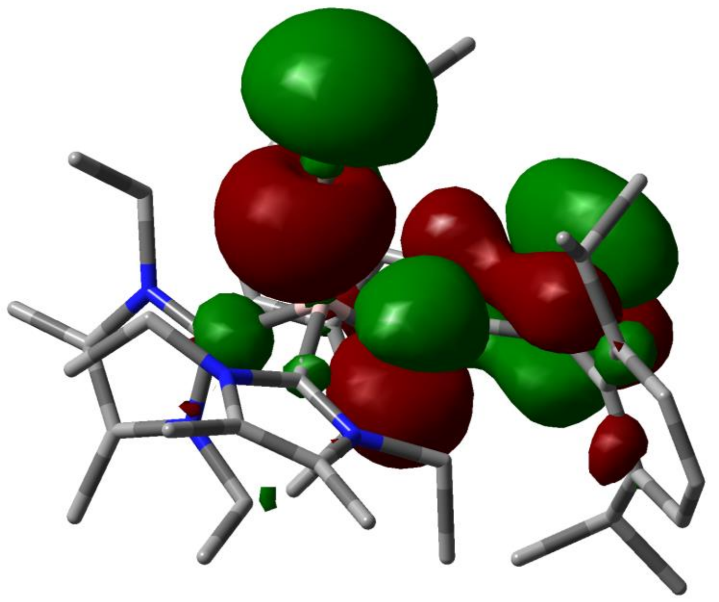

Supplementary Figure 21. HOMO−3 of **3** at B97-D/6-31G(d)[Al: cc-pVTZ, Te: cc-pVTZ-PP] level of theory (stick model). Te: yellow, Al: pink, N: blue, C: grey. Hydrogen atoms have been omitted for clarity.

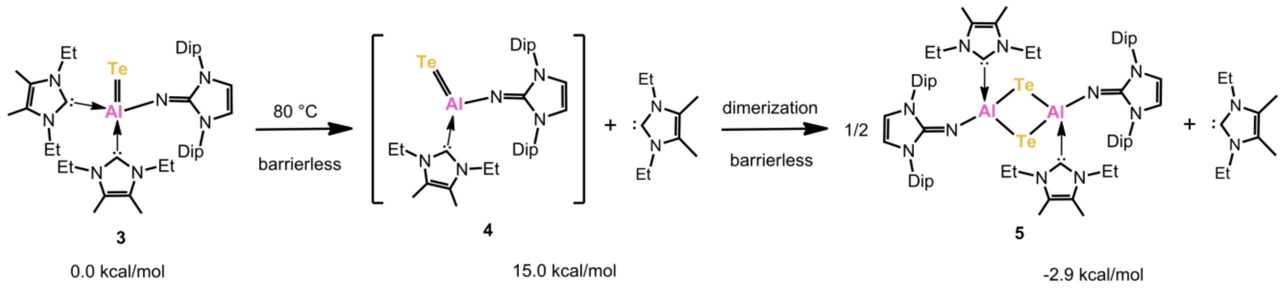

Supplementary Figure 22. Calculated mechanism of the reaction from **3** to **5** at ωB97X-D/cc-pVTZ[Te: cc-pVTZ-PP](SMD=benzene)//B97-D/6-31G(d)[Al: cc-pVTZ, Te: cc-pVTZ-PP] level of theory.

Supplementary Tables

Supplementary Table 1. Bond analyses to **3**, **4**, **5** and to selected sterically reduced model compounds (L^{Me} = 1,3,4,5-tetramethyl-imidazolin-2-ylidene).

	Al–Te bond length [Å]	Wiberg Bond Index	NPA charge Al	NPA charge Te	NRT
$[Me_3AlTe]^{2-}$	2.674	0.94	1.40	-1.35	Al−Te: 100.0%
$[Me_2AlTe]^-$	2.475	1.41	1.26	-1.02	Al=Te: 100.0%
$[Me_2(L^{Me})AlTe]^-$	2.562	1.12	1.26	-1.11	Al=Te: 65.5%<br>Al−Te: 34.5%
$Me(L^{Me})_2AlTe$	2.508	1.20	1.16	-0.90	Al=Te: 78.2%<br>Al−Te: 21.8%
$Me(L^{Me})AlTe$	2.422	1.61	1.11	-0.77	Al≡Te: 8.9%<br>Al=Te: 91.1%
MeAlTe	2.355	2.09	1.12	-0.62	Al≡Te: 100.0%
**3**	2.505	1.20	1.24	-0.95	Al=Te: 76.7%<br>Al−Te: 23.3%
**4**	2.428	1.52	1.22	-0.84	Al≡Te: 5.1%<br>Al=Te: 94.9%
**5**	2.657	0.75	1.21	-0.79	Al−Te: 100.0%

Supplementary Table 2. Cartesian geometry of **2** at B97-D/6-31G(d)[Al: cc-pVTZ, Te: cc-pVTZ-PP] level of theory in [Å].

C	3.464488	2.402522	0.972753
C	3.572677	1.616934	-0.198424
C	3.654791	2.186979	-1.495108
C	3.559932	3.588535	-1.583971
C	3.404619	4.384891	-0.441274
C	3.372429	3.796970	0.825459
N	3.616691	0.184600	-0.040267
C	2.506641	-0.604577	0.281203
N	3.049724	-1.826970	0.678093
C	4.447989	-1.756527	0.667929
C	4.795827	-0.517527	0.241594
N	1.244626	-0.268188	0.275031
Al	-0.373836	-1.333052	0.138188
C	2.324906	-2.926451	1.262741
C	1.888532	-2.811876	2.607601
C	1.249196	-3.923812	3.180779
C	1.044128	-5.097699	2.445375
C	1.470260	-5.178529	1.116479
C	2.118044	-4.095396	0.491645
C	2.107289	-1.528991	3.411118
C	3.537678	-1.471378	3.998166
C	2.583514	-4.207175	-0.959566
C	1.638020	-5.069379	-1.824969
C	3.889872	1.329680	-2.740257
C	3.482247	2.025558	-4.055140
C	3.448370	1.797224	2.372802
C	2.105408	2.070154	3.079353
C	1.076587	-1.331506	4.540331
C	4.016484	-4.790162	-1.038027
C	5.379821	0.914750	-2.851120
C	4.634546	2.318619	3.215741
Al	0.309443	1.268695	-0.376096
N	-1.276680	0.366487	0.311412
C	-2.510958	0.785521	0.408412
N	-2.929788	2.110269	0.567742
C	-4.324779	2.165961	0.672919
C	-4.794912	0.899283	0.560165
N	-3.694917	0.048644	0.395976
C	-2.084565	3.159533	1.072814
C	-1.587716	4.134890	0.180807
C	-0.798186	5.164318	0.721517
C	-0.487833	5.192122	2.087056
C	-0.975849	4.197751	2.944457
C	-1.799048	3.166779	2.457288

C	-1.881941	4.048413	-1.314102
C	-3.215351	4.756208	-1.651904
C	-2.342710	2.102122	3.408076
C	-3.097742	2.733426	4.599355
C	-3.795257	-1.367053	0.171203
C	-4.288714	-1.819134	-1.080059
C	-4.314816	-3.209012	-1.288527
C	-3.897155	-4.099623	-0.290911
C	-3.479132	-3.623942	0.956320
C	-3.427115	-2.243113	1.221676
C	-4.815177	-0.838483	-2.135040
C	-3.686523	-0.051752	-2.837673
C	-3.041422	-1.700450	2.601755
C	-4.278586	-1.126185	3.337010
C	-5.718651	-1.503543	-3.194254
C	-2.362696	-2.739881	3.514111
C	-1.212573	1.174971	3.901348
C	-0.737456	4.599458	-2.189563
H	6.023523	1.809677	-2.893138
H	3.599053	4.064604	-2.563095
H	3.314625	5.468680	-0.544034
H	3.257340	4.417540	1.715921
H	5.596183	2.098445	2.724784
H	4.630342	1.837743	4.207958
H	4.566100	3.408741	3.363103
H	4.750231	-4.152235	-0.524456
H	4.325286	-4.885412	-2.092200
H	4.045346	-5.792126	-0.577466
H	5.046579	-2.601556	0.981070
H	1.741873	-6.141840	-1.586845
H	1.890167	-4.931177	-2.887038
H	3.516973	1.292860	-4.875483
H	4.179359	2.845179	-4.302088
H	5.533970	0.337116	-3.777554
H	5.703342	0.290389	-2.008141
H	5.763031	-0.048037	0.119571
H	1.289217	-6.090729	0.547881
H	0.540018	-5.948835	2.908886
H	0.893742	-3.868292	4.209213
H	2.589575	-3.195646	-1.391957
H	0.587037	-4.778616	-1.680943
H	1.982995	-0.683521	2.722998
H	1.191937	-0.322946	4.964815
H	1.226215	-2.057785	5.356630
H	0.050685	-1.434684	4.162841
H	4.304340	-1.512318	3.211165
H	3.698460	-2.315255	4.689673
H	3.673939	-0.532102	4.559718

H	3.278628	0.420246	-2.636997
H	2.460377	2.425951	-4.001089
H	3.568088	0.710189	2.294381
H	1.271040	1.653817	2.501140
H	1.928849	3.151332	3.191600
H	2.105317	1.608838	4.080466
H	-4.828621	3.108726	0.844162
H	-5.801695	0.501828	0.575615
H	-0.724018	4.220682	4.006424
H	0.145416	5.989270	2.483902
H	-0.388152	5.926713	0.058939
H	-3.066034	1.475879	2.869838
H	-0.711431	0.690821	3.051571
H	-0.459420	1.743064	4.469509
H	-1.625071	0.391591	4.557570
H	-3.911346	3.389189	4.249980
H	-3.532374	1.939869	5.228953
H	-2.419584	3.333040	5.228200
H	-1.998208	2.983312	-1.570879
H	-0.926893	4.332639	-3.240892
H	-0.674095	5.699275	-2.124553
H	0.230355	4.173166	-1.885135
H	-4.057509	4.305906	-1.103738
H	-3.160357	5.825993	-1.387948
H	-3.423216	4.674167	-2.731449
H	-3.172507	-4.331354	1.725543
H	-3.910517	-5.174481	-0.484802
H	-4.659700	-3.602542	-2.243587
H	-2.325215	-0.879441	2.449870
H	-1.478178	-3.178856	3.032278
H	-2.049502	-2.249976	4.449633
H	-3.064837	-3.547485	3.782199
H	-3.972703	-0.720148	4.315877
H	-4.763383	-0.319154	2.771116
H	-5.019421	-1.923852	3.513064
H	-5.441092	-0.100261	-1.607681
H	-6.532849	-2.086507	-2.733282
H	-6.162724	-0.723193	-3.831910
H	-5.136369	-2.174680	-3.846503
H	-3.014951	0.449277	-2.129573
H	-3.066066	-0.730315	-3.439336
H	-4.126721	0.712928	-3.499424
Te	-0.336539	-1.918433	-2.440138
Te	0.045896	0.825104	-2.974476
H	-0.637769	-2.494463	1.167209
H	0.721312	2.724505	0.050007

Supplementary Table 3. Cartesian geometry of **3** at B97-D/6-31G(d)[Al: cc-pVTZ, Te: cc-pVTZ-PP] level of theory in [Å].

Te	-0.981817	0.861783	-3.013694
Al	-0.492295	0.722742	-0.561186
N	0.777019	-0.365817	0.177041
N	0.489113	-2.765905	-0.260469
N	2.542295	-1.937807	-0.259847
N	-3.376686	0.128954	0.561372
N	-2.023120	0.763729	2.115059
N	-0.753637	3.781910	-0.173143
N	1.275615	3.187304	0.256916
C	1.197277	-1.541708	-0.087473
C	1.380808	-3.815947	-0.516557
H	1.029197	-4.831715	-0.638609
C	2.640469	-3.308308	-0.511641
H	3.601208	-3.781232	-0.677724
C	-0.906711	-2.967859	-0.006355
C	-1.764986	-3.295484	-1.087523
C	-3.109209	-3.596043	-0.791128
H	-3.783991	-3.859553	-1.606089
C	-3.594320	-3.541097	0.518673
H	-4.641565	-3.777787	0.725239
C	-2.736072	-3.189158	1.569450
H	-3.126608	-3.150350	2.586015
C	-1.376791	-2.914607	1.335728
C	-1.268334	-3.368360	-2.531592
H	-0.353660	-2.761126	-2.601542
C	-2.280913	-2.792121	-3.545607
H	-2.544904	-1.755830	-3.291799
H	-3.190821	-3.414543	-3.607377
H	-1.817518	-2.777729	-4.544542
C	-0.944306	-4.831307	-2.927086
H	-0.177169	-5.284765	-2.282884
H	-0.581024	-4.864447	-3.967486
H	-1.854713	-5.451343	-2.861242
C	-0.420295	-2.627423	2.496033
H	0.203101	-1.772668	2.199849
C	-1.133187	-2.249544	3.809574
H	-1.888544	-1.469598	3.646813
H	-0.389201	-1.885665	4.536535
H	-1.637239	-3.122708	4.257487
C	0.505599	-3.837978	2.774716
H	1.168168	-3.610759	3.626394
H	1.134056	-4.087286	1.910124
H	-0.098970	-4.722726	3.036882
C	3.659957	-1.054469	-0.128739

C	3.960275	-0.162237	-1.186669
C	5.101547	0.650260	-1.043119
H	5.361211	1.346719	-1.840994
C	5.901320	0.583005	0.105708
H	6.787850	1.216613	0.190246
C	5.559496	-0.281344	1.154649
H	6.173676	-0.306695	2.055949
C	4.425042	-1.109044	1.061957
C	3.055588	-0.073149	-2.412898
H	2.012959	-0.158934	-2.071836
C	3.316902	-1.242442	-3.391235
H	3.203330	-2.217080	-2.892485
H	4.335840	-1.178261	-3.811304
H	2.589313	-1.189301	-4.216986
C	3.144729	1.272982	-3.156116
H	3.011087	2.118780	-2.462405
H	2.328416	1.317264	-3.891890
H	4.109964	1.391508	-3.679635
C	3.954335	-1.949623	2.250293
H	3.424826	-2.832967	1.865540
C	2.931623	-1.123851	3.070064
H	2.483435	-1.746766	3.862186
H	2.134229	-0.739801	2.416664
H	3.434685	-0.264823	3.544261
C	5.095146	-2.453883	3.156623
H	5.857410	-3.000773	2.578658
H	4.684743	-3.129901	3.924054
H	5.591815	-1.620913	3.681091
C	-2.092215	0.527629	0.768322
C	-4.106540	0.108118	1.749142
C	-3.250147	0.523346	2.740598
C	-5.523291	-0.365012	1.829786
H	-5.862418	-0.362298	2.875697
H	-5.609600	-1.394145	1.442458
H	-6.209180	0.268802	1.244168
C	-3.488692	0.713490	4.207042
H	-4.464330	0.288278	4.483265
H	-3.491182	1.779740	4.491678
H	-2.717177	0.211684	4.813736
C	-3.935767	-0.246049	-0.751986
H	-4.519551	-1.163576	-0.601936
H	-3.084694	-0.467717	-1.410869
C	-4.777377	0.877394	-1.366053
H	-5.220704	0.517203	-2.307173
H	-4.131551	1.731604	-1.610645
H	-5.588799	1.203493	-0.694828
C	-0.789988	1.136134	2.828160
H	0.044757	0.883737	2.163293

H	-0.717359	0.484298	3.710957
C	-0.757831	2.613005	3.244718
H	0.186173	2.818791	3.773140
H	-1.592253	2.855957	3.920544
H	-0.814110	3.267037	2.365904
C	0.044964	2.674572	-0.044525
C	-0.032438	4.961542	0.018574
C	1.259172	4.584775	0.291604
C	-0.638613	6.323573	-0.116897
H	0.122924	7.093200	0.073823
H	-1.466940	6.479053	0.595090
H	-1.041359	6.487944	-1.131203
C	2.478910	5.420069	0.528312
H	2.247633	6.479270	0.344911
H	3.298155	5.129025	-0.150483
H	2.859715	5.328440	1.559610
C	-2.164514	3.741889	-0.583272
H	-2.294837	2.813005	-1.160062
H	-2.324944	4.574856	-1.283538
C	-3.137119	3.837589	0.600724
H	-4.171371	3.837403	0.221432
H	-2.979398	4.764818	1.175853
H	-3.017166	2.982241	1.278304
C	2.481670	2.373433	0.483064
H	3.295720	2.800485	-0.119567
H	2.260932	1.364970	0.115943
C	2.874710	2.305566	1.963446
H	3.791805	1.706206	2.055626
H	2.081342	1.812259	2.542611
H	3.055976	3.304186	2.391144

Supplementary Table 4. Cartesian geometry of **4** at B97-D/6-31G(d)[Al: cc-pVTZ, Te: cc-pVTZ-PP] level of theory in [Å].

C	4.237839	1.722880	-0.152963
C	3.633669	0.454105	-0.329176
C	3.792768	-0.314185	-1.509047
C	4.611092	0.223258	-2.520596
C	5.241074	1.464355	-2.362273
C	5.053726	2.209041	-1.190811
N	2.824401	-0.065320	0.733413
C	1.427083	-0.172954	0.671670
N	1.088996	-0.771384	1.905168
C	2.239513	-1.028393	2.658526
C	3.306309	-0.585487	1.940823
N	0.650678	0.213388	-0.270943
Al	-0.913740	-0.319552	-0.956090
Te	-1.710541	-2.188371	-2.286260
C	-0.252616	-1.139234	2.231898
C	-0.626141	-2.502308	2.156434
C	-1.960403	-2.833544	2.461422
C	-2.888211	-1.843651	2.799154
C	-2.499550	-0.496190	2.850897
C	-1.175088	-0.113568	2.575612
C	0.378071	-3.592778	1.788342
C	-0.185251	-4.614694	0.777656
C	-0.716809	1.344195	2.666237
C	-1.873651	2.362531	2.684840
C	3.112438	-1.676646	-1.664930
C	3.836585	-2.755824	-0.822854
C	3.939339	2.559895	1.093924
C	2.537286	3.205129	0.963982
C	-2.172351	1.280888	-0.705582
N	-3.505424	1.261945	-0.416694
C	-4.024362	2.554168	-0.336835
C	-2.977234	3.409443	-0.592897
N	-1.859332	2.606301	-0.803626
C	-4.311942	0.034809	-0.257346
C	-5.120755	-0.274440	-1.522255
C	-5.443372	2.848292	0.038319
C	-2.924984	4.904814	-0.617332
C	-0.525655	3.101533	-1.191017
C	-0.379114	3.173718	-2.717203
C	0.865969	-4.313725	3.068677
C	0.179033	1.570893	3.909060
C	2.990922	-2.146087	-3.128112
C	4.997593	3.640408	1.393437
H	2.182251	-1.497064	3.632985

H	4.367421	-0.597474	2.158499
H	-2.275635	-3.875787	2.404034
H	-3.923078	-2.118876	3.015518
H	-3.236548	0.264009	3.110806
H	1.248534	-3.112459	1.318860
H	-0.556021	-4.109388	-0.127681
H	-1.007338	-5.203208	1.217864
H	0.613668	-5.319427	0.493386
H	1.321905	-3.610600	3.784179
H	1.613535	-5.081463	2.809116
H	0.020235	-4.811117	3.572359
H	-0.115553	1.548733	1.768734
H	-2.567924	2.208896	1.847963
H	-1.461848	3.382067	2.613375
H	-2.444638	2.298988	3.626748
H	0.522652	2.618901	3.935577
H	1.065076	0.921353	3.898688
H	-0.392723	1.372297	4.831256
H	4.754726	-0.336296	-3.444849
H	5.876350	1.856811	-3.159924
H	5.541093	3.178894	-1.086539
H	2.085594	-1.579166	-1.286398
H	3.872545	-2.485021	0.243051
H	4.870443	-2.894975	-1.182713
H	3.305082	-3.717306	-0.917931
H	2.534802	-1.368446	-3.759893
H	2.346025	-3.036578	-3.167370
H	3.975275	-2.411329	-3.551794
H	3.903049	1.885637	1.963540
H	2.280967	3.755264	1.885647
H	1.770210	2.440770	0.780812
H	2.526359	3.914708	0.118936
H	6.011643	3.211825	1.437374
H	4.775001	4.116992	2.361450
H	4.990753	4.430236	0.624162
H	-5.598812	3.935271	0.088263
H	-5.691939	2.423957	1.026276
H	-6.158352	2.431309	-0.688832
H	-3.926902	5.318658	-0.435969
H	-2.569204	5.284682	-1.589704
H	-2.248657	5.298217	0.161183
H	-4.957036	0.180226	0.622762
H	-3.613263	-0.788323	-0.054349
H	-5.731935	-1.173670	-1.348394
H	-4.425201	-0.488276	-2.347450
H	-5.788212	0.558752	-1.794240
H	0.207449	2.403328	-0.768608
H	-0.390681	4.087402	-0.724103

H	0.628845	3.535542	-2.973695
H	-1.125068	3.851804	-3.162324
H	-0.510299	2.171781	-3.155283

Supplementary Table 5. Cartesian geometry of **5** at B97-D/6-31G(d)[Al: cc-pVTZ, Te: cc-pVTZ-PP] level of theory in [Å].

C	2.538000	3.635884	1.928828
C	3.057248	3.652662	0.609813
C	2.503691	4.461942	-0.409341
C	1.479817	5.360607	-0.051518
C	0.975149	5.389508	1.252720
C	1.482656	4.515055	2.225460
N	4.157017	2.797346	0.280530
C	3.967702	1.428271	-0.058619
N	5.209543	1.089149	-0.682978
C	6.074003	2.194176	-0.689939
C	5.425822	3.237869	-0.107692
N	2.950279	0.720146	0.150108
Al	1.629080	-0.475368	0.270454
Te	-0.035509	-0.252248	-1.762052
C	5.476641	-0.158993	-1.323637
C	4.810979	-0.486821	-2.540171
C	5.115056	-1.725047	-3.133510
C	6.048910	-2.602649	-2.562508
C	6.695684	-2.256404	-1.373042
C	6.416376	-1.035143	-0.729376
C	3.870385	0.506211	-3.226933
C	2.963634	-0.131794	-4.295519
C	7.144187	-0.672007	0.564041
C	7.086141	-1.814407	1.596145
C	2.987983	4.383193	-1.858420
C	3.692953	5.688606	-2.286120
C	3.138627	2.703716	2.979657
C	2.209899	2.459506	4.184500
C	2.672741	-2.261739	0.502237
N	2.967625	-3.230609	-0.405350
C	3.804951	-4.207247	0.132823
C	4.056934	-3.827454	1.428344
N	3.361398	-2.630126	1.625357
C	2.409703	-3.294572	-1.761281
C	1.160779	-4.183530	-1.788295
C	4.334881	-5.356126	-0.664011
C	4.869564	-4.514253	2.483713
C	3.405416	-1.846497	2.873685
C	2.606031	-2.503942	4.004938
C	4.679632	1.651212	-3.889640
C	8.614075	-0.283077	0.279530
C	1.823686	4.042213	-2.816863
C	4.510060	3.247211	3.449117
H	7.070689	2.120868	-1.107278

H	5.740840	4.261209	0.059988
H	4.623973	-2.002348	-4.066676
H	6.282097	-3.548966	-3.057835
H	7.427558	-2.936501	-0.933405
H	3.214278	0.937410	-2.457468
H	2.407355	-0.995358	-3.904089
H	3.547869	-0.454489	-5.174833
H	2.226882	0.613289	-4.630053
H	5.307039	2.192126	-3.168292
H	3.987007	2.373236	-4.351134
H	5.330994	1.245070	-4.682617
H	6.638632	0.200067	1.003319
H	6.050496	-2.145297	1.725958
H	7.484926	-1.473021	2.565655
H	7.681915	-2.684388	1.274001
H	9.127287	-0.006765	1.215962
H	8.674751	0.569652	-0.413357
H	9.153640	-1.131371	-0.173970
H	1.074258	6.031683	-0.812599
H	0.168559	6.077950	1.514566
H	1.049071	4.516158	3.225904
H	3.714131	3.564836	-1.935459
H	4.546579	5.911719	-1.626895
H	2.995780	6.542579	-2.243956
H	4.064203	5.597930	-3.320451
H	1.371130	3.076691	-2.546784
H	2.191286	3.987303	-3.854276
H	1.038312	4.813906	-2.778310
H	3.302822	1.728562	2.495674
H	2.655910	1.694388	4.840970
H	1.224523	2.096302	3.849072
H	2.078944	3.374737	4.787962
H	5.205119	3.364048	2.603745
H	4.961753	2.551632	4.176697
H	4.386119	4.228081	3.939965
H	5.008775	-5.962678	-0.042049
H	4.904438	-4.990819	-1.535581
H	3.530097	-6.011180	-1.036924
H	5.566860	-5.222123	2.012264
H	4.236499	-5.078595	3.189735
H	5.463776	-3.799663	3.072862
H	3.208127	-3.644034	-2.430032
H	2.138134	-2.274886	-2.052552
H	0.772332	-4.265225	-2.815472
H	0.383283	-3.723284	-1.160888
H	1.376236	-5.196513	-1.410375
H	3.014175	-0.846984	2.646531
H	4.463151	-1.736055	3.155977

H	2.659672	-1.868623	4.903222
H	3.009057	-3.497459	4.255218
H	1.550688	-2.596824	3.710436
Te	-0.014665	-0.432233	2.327183
Al	-1.610851	0.163916	0.319694
N	-3.251938	-0.505248	0.125391
C	-2.106873	2.138398	0.747582
N	-2.865920	2.535969	1.815839
C	-3.104687	3.911838	1.786606
C	-2.502041	4.380908	0.645447
N	-1.896391	3.279740	0.042668
C	-1.180759	3.334463	-1.240324
C	-2.441327	5.762516	0.073501
C	-3.869973	4.645730	2.842857
C	-3.382880	1.637776	2.861514
H	-3.277792	0.612249	2.489690
C	-2.653886	1.819240	4.199125
H	-4.461498	1.843450	2.961795
H	-0.347563	2.623254	-1.181995
C	-2.109976	2.992760	-2.409724
H	-0.763602	4.343066	-1.335033
H	-3.098589	6.433425	0.645188
H	-1.418414	6.174706	0.110387
H	-2.767036	5.776687	-0.980064
H	-4.133391	5.650248	2.480380
H	-4.805164	4.122768	3.097184
H	-3.286276	4.761787	3.772027
H	-3.013732	1.067863	4.918654
H	-1.574322	1.669469	4.052684
H	-2.831568	2.820365	4.622116
H	-1.556889	3.087694	-3.357708
H	-2.467320	1.958637	-2.320896
H	-2.989613	3.654523	-2.433516
C	-4.303099	-1.174641	-0.055120
N	-4.639331	-2.479417	0.401198
C	-5.909737	-2.847391	-0.060744
C	-6.410774	-1.811911	-0.785891
N	-5.444236	-0.801157	-0.809764
C	-5.492857	0.388692	-1.596107
H	-7.366029	-1.685611	-1.281052
H	-6.338043	-3.811556	0.186646
C	-3.606525	-3.383266	0.808854
C	-4.893638	0.388084	-2.879409
C	-5.033315	1.548267	-3.662756
C	-5.710285	2.673540	-3.171913
C	-6.233162	2.672416	-1.872003
C	-6.122536	1.531330	-1.055266
C	-4.113698	-0.829931	-3.379480

H	-4.588348	1.579602	-4.657676
H	-5.810263	3.563261	-3.798671
H	-6.722831	3.567491	-1.485175
C	-6.654048	1.506992	0.377211
C	-3.181410	-3.375534	2.159154
C	-2.133095	-4.237530	2.521331
C	-1.535925	-5.081635	1.573925
C	-1.963514	-5.062260	0.241512
C	-2.992091	-4.195507	-0.174767
C	-3.868684	-2.458287	3.169053
H	-1.764431	-4.238972	3.547223
H	-0.718268	-5.741721	1.873562
H	-1.485228	-5.714913	-0.492290
C	-3.426363	-4.159613	-1.640272
H	-3.623380	-1.285803	-2.507311
C	-5.060858	-1.877259	-4.010886
C	-2.995737	-0.469708	-4.378981
H	-4.478756	-2.747867	-4.358193
H	-5.813080	-2.232525	-3.290356
H	-5.582342	-1.441980	-4.880344
H	-2.398600	-1.369570	-4.591595
H	-3.410440	-0.103508	-5.333979
H	-2.319839	0.293657	-3.969098
H	-6.043138	0.782419	0.936841
C	-6.519109	2.871112	1.088326
C	-8.120071	1.019131	0.413495
H	-6.726008	2.749683	2.164830
H	-5.505038	3.278561	0.963826
H	-7.239322	3.608140	0.696134
H	-8.497471	1.001447	1.450290
H	-8.761898	1.692858	-0.179025
H	-8.205566	0.004567	-0.002528
H	-4.026928	-1.491585	2.665669
C	-5.256970	-3.025605	3.557357
C	-3.037444	-2.199179	4.438383
H	-5.752891	-2.355179	4.280541
H	-5.909437	-3.128233	2.678479
H	-5.144094	-4.016385	4.029067
H	-3.537573	-1.433009	5.052965
H	-2.954476	-3.112875	5.052574
H	-2.024758	-1.850507	4.185511
H	-4.064084	-3.280817	-1.797065
C	-2.233536	-4.012210	-2.609377
C	-4.266424	-5.409439	-1.987891
H	-4.603206	-5.362623	-3.037323
H	-3.667076	-6.326113	-1.855551
H	-5.154066	-5.482761	-1.339753
H	-2.609559	-3.953292	-3.645068

H	-1.662637	-3.096099	-2.394389
H	-1.550523	-4.875536	-2.551046

Supplementary Table 6. Crystallographic data to **2**.

Empirical formula	$C_{54}H_{72}Al_2N_6Te_2$	(the two Al-bond H atoms were not identified)
Formula weight	1114.34	
Temperature	150.00(10) K	
Wavelength	1.54184 Å	
Crystal system	Monoclinic	
Space group	$P2_1/n$	
Unit cell dimensions	a = 12.4329(2) Å	α = 90°.
	b = 22.9150(4) Å	β = 92.861(2)°.
	c = 19.4190(5) Å	γ = 90°.
Volume	5525.58(19) $Å^3$	
Z	4	
Density (calculated)	1.340 Mg/m^3	
Absorption coefficient	8.932 mm^{-1}	
F(000)	2272	
Crystal colour, shape	blue, plate	
Crystal size	0.34 x 0.09 x 0.05 mm^3	
Theta range for data collection	2.99 to 67.50°.	
Index ranges	-14<=h<=14, -26<=k<=27, -22<=l<=23	
Reflections collected	35751	
Independent reflections	9958 [R(int) = 0.0805]	
Completeness to theta = 67.50°	100.0 %	
Absorption correction	Semi-empirical from equivalents	
Max. and min. transmission	0.6841 and 0.1522	
Refinement method	Full-matrix least-squares on F^2	
Data / restraints / parameters	9958 / 6 / 593	
Goodness-of-fit on F^2	1.059	
Final R indices [I>2sigma(I)]	R1 = 0.1777, wR2 = 0.4134	
R indices (all data)	R1 = 0.1839, wR2 = 0.4164	
Largest diff. peak and hole	11.807 and -2.448 e.$Å^{-3}$	

Supplementary Table 7. Crystallographic data to **3**.

Empirical formula	$C_{45}H_{68}AlN_7Te$	
Formula weight	861.64	
Temperature	150.00(10) K	
Wavelength	1.54184 Å	
Crystal system	Monoclinic	
Space group	$P2_1/c$	
Unit cell dimensions	a = 12.2033(4) Å	α = 90°.
	b = 19.6490(7) Å	β = 95.069(3)°.
	c = 19.4970(6) Å	γ = 90°.
Volume	4656.8(3) $Å^3$	
Z	4	
Density (calculated)	1.229 Mg/m^3	
Absorption coefficient	5.499 mm^{-1}	
F(000)	1808	
Crystal colour, shape	Yellow, plate	
Crystal size	0.36 x 0.21 x 0.09 mm^3	
Theta range for data collection	3.20 to 67.49°.	
Index ranges	-11<=h<=14, -23<=k<=23, -23<=l<=18	
Reflections collected	16375	
Independent reflections	8090 [R(int) = 0.0506]	
Completeness to theta = 67.49°	96.2 %	
Absorption correction	Semi-empirical from equivalents	
Max. and min. transmission	0.6374 and 0.2422	
Refinement method	Full-matrix least-squares on F^2	
Data / restraints / parameters	8090 / 0 / 503	
Goodness-of-fit on F^2	1.064	
Final R indices [I>2sigma(I)]	R1 = 0.0529, wR2 = 0.1348	
R indices (all data)	R1 = 0.0717, wR2 = 0.1709	
Largest diff. peak and hole	2.328 and -1.509 e.$Å^{-3}$	

Supplementary Table 8. Crystallographic data to **5**.

Empirical formula	$C_{72}H_{104}Al_2N_{10}Te_2$	
Formula weight	1418.81	
Temperature	150.00(10) K	
Wavelength	1.54184 Å	
Crystal system	Triclinic	
Space group	*P*-1	
Unit cell dimensions	a = 10.4285(6) Å	α = 107.012(5)°.
	b = 12.9619(8) Å	β = 101.576(5)°.
	c = 15.2266(7) Å	γ = 104.722(5)°.
Volume	1817.43(18) $Å^3$	
Z	1	
Density (calculated)	1.296 Mg/m^3	
Absorption coefficient	6.917 mm^{-1}	
F(000)	736	
Crystal colour, shape	Colourless, rod	
Crystal size	0.23 x 0.19 x 0.13 mm^3	
Theta range for data collection	3.18 to 67.50°.	
Index ranges	-11<=h<=12, -15<=k<=15, -18<=l<=13	
Reflections collected	11605	
Independent reflections	6533 [R(int) = 0.0879]	
Completeness to theta = 67.50°	99.5 %	
Absorption correction	Semi-empirical from equivalents	
Max. and min. transmission	0.4621 and 0.2991	
Refinement method	Full-matrix least-squares on F^2	
Data / restraints / parameters	6533 / 0 / 400	
Goodness-of-fit on F^2	1.084	
Final R indices [I>2sigma(I)]	R1 = 0.0584, wR2 = 0.1504	
R indices (all data)	R1 = 0.0658, wR2 = 0.1618	
Largest diff. peak and hole	1.846 and -1.627 e.$Å^{-3}$	

Supplementary Methods

NMR-spectroscopic characterization of $L^{Et}(H_2)$.[1]

^{1}H NMR (500.1 MHz, C_6D_6): δ = 3.83 (s, 2 H, NCH_2N), 2.57 (q, J_{HH} = 7 Hz, 4 H, CH_2CH$_3$), 1.57 (s, 6 H, CCH_3), 0.98 (t, J_{HH} = 7 Hz, 6 H, CH$_2$CH_3); **^{13}C{^{1}H} NMR (125.8 MHz, C_6D_6):** δ = 123.0 (*C*CH$_3$), 76.5 (N*C*H$_2$N), 46.0 (*C*H$_2$CH$_3$), 13.8 (CH$_2$*C*H$_3$), 10.3 (C*C*H$_3$).

Crystallographic Methods

Data for the single crystal structure determination of **2**, **3** and **5** were collected on an Agilent SuperNova diffractometer, equipped with a CCD area Atlas detector and a mirror monochromator utilizing CuK_{α} radiation (λ = 1.54184 Å).

The crystal structures were solved by Direct Methods and refined on $\mathbf{F}^2$ using full-matrix least squares with SHELXL-97 (G. M. Sheldrick, SHELXL-97, *Program for refinement of crystal structures*, University of Göttingen, Germany, 1997). The positions of the H atoms at the carbon atoms were calculated by standard methods.

Supplementary Reference

1. Sindlinger, P. C., Wesemann, L. Hydrogen abstraction from organotin di- and trihydrides by *N*-heterocyclic carbenes: a new method for the preparation of NHC adducts to tin(II) species and observation of an isomer of a hexastannane derivative [R_6Sn_6]. *Chem. Sci.* **5,** 2739–2746 (2014).
